# The Systematic Functional Analysis of *Plasmodium* Protein Kinases Identifies Essential Regulators of Mosquito Transmission

**DOI:** 10.1016/j.chom.2010.09.006

**Published:** 2010-10-21

**Authors:** Rita Tewari, Ursula Straschil, Alex Bateman, Ulrike Böhme, Inna Cherevach, Peng Gong, Arnab Pain, Oliver Billker

**Affiliations:** 1 Institute of Genetics, QMC, University of Nottingham, Nottingham NG7 2UH, UK; 2 Division of Cell & Molecular Biology, Imperial College London, Exhibition Road, London SW7 2AZ, UK; 3 The Wellcome Trust Sanger Institute, Hinxton, Cambridge CB10 1SA, UK; 4 Computational Bioscience Research Center, Chemical Life Sciences and Engineering Division, King Abdullah University of Science and Technology, Thuwal 23955-6900, Kingdom of Saudi Arabia

## Abstract

Although eukaryotic protein kinases (ePKs) contribute to many cellular processes, only three *Plasmodium falciparum* ePKs have thus far been identified as essential for parasite asexual blood stage development. To identify pathways essential for parasite transmission between their mammalian host and mosquito vector, we undertook a systematic functional analysis of ePKs in the genetically tractable rodent parasite *Plasmodium berghei*. Modeling domain signatures of conventional ePKs identified 66 putative *Plasmodium* ePKs. Kinomes are highly conserved between *Plasmodium* species. Using reverse genetics, we show that 23 ePKs are redundant for asexual erythrocytic parasite development in mice. Phenotyping mutants at four life cycle stages in *Anopheles stephensi* mosquitoes revealed functional clusters of kinases required for sexual development and sporogony. Roles for a putative SR protein kinase (SRPK) in microgamete formation, a conserved regulator of clathrin uncoating (GAK) in ookinete formation, and a likely regulator of energy metabolism (SNF1/KIN) in sporozoite development were identified.

## Highlights

► Domain signature modeling identifies 66 putative *Plasmodium* eukaryotic protein kinases ► The complement of protein kinases is largely conserved between *Plasmodium* species ► 23 protein kinase genes are redundant for *P. berghei* asexual erythrocytic development in mice ► 13 mutants reveal essential kinase gene functions in mosquito transmission

## Introduction

Malaria parasites, transmitted through the bites of infected *Anopheles* mosquitoes, cause almost 250 million infections, leading to nearly a million deaths annually (WHO, 2008). Blocking parasite transmission is recognized as an important element in the global fight to control malaria (Wells et al., 2009), yet little is known about the molecular pathways that regulate host-pathogen interactions and initiate the cellular differentiation processes that enable parasites to alternate between host and vector. Key roles for protein kinases as regulators of parasite development in the mosquito have recently begun to emerge from studies in *P. falciparum*, the most lethal malaria parasite of humans, and *P. berghei*, a species that infects rodents and in which mosquito stages are particularly accessible to experimentation (Billker et al., 2009; Doerig et al., 2008).

All pathology of malaria is caused by parasites replicating asexually in red blood cells. In contrast, transmission to the mosquito relies entirely on the ingestion of developmentally arrested sexual precursor stages, the gametocytes, into the blood meal of a vector. The switch to sexual development is in part environmentally controlled, but the host factors involved and the parasite pathways through which they act have remained enigmatic. In the blood meal, a drop in temperature, together with a small rise in pH and xanthurenic acid from the mosquito, trigger the rapid differentiation of gametocytes into gametes (Billker et al., 1998). Activation of developmentally arrested gametocytes involves the secondary messengers Ca^2+^ and cyclic guanosine monophosphate (cGMP) and relies on the cGMP-dependent protein kinase (PKG) (McRobert et al., 2008). Once activated, male gametocytes require a Ca^2+^-dependent protein kinase, CDPK4, to initiate replication of their genome (Billker et al., 2004). Following the completion of mitosis, an atypical mitogen-activated protein kinase, MAP-2, becomes essential for the explosive release of eight flagellated microgametes in a process termed exflagellation (Tewari et al., 2005a). Fertilization is followed by meiosis, which involves its own set of cellular regulators, including two stage-specifically expressed NIMA-related protein kinases, Nek-2 and Nek-4 (Reininger et al., 2009). Within 24 hr, zygotes transform into ookinetes, which move actively through the blood meal, a process requiring CDPK3 and a cGMP signaling pathway (Billker et al., 2009; Moon et al., 2009). Ookinetes penetrate the mosquito-derived peritrophic matrix that encloses the blood bolus, cross the epithelial monolayer of the mosquito midgut, and transform into oocysts. These grow and replicate until each cyst actively releases thousands of sporozoites into the hemocoel. Sporozoites enter the salivary glands and are injected into another host when the mosquito bites again. In the liver, sporozoites respond to cell sulfation levels and change from migrating through cells to invading a parenchymal cell by entering a parasitophorous vacuole, a behavioral switch promoted in vitro by *cdpk6* (Coppi et al., 2007). Eventually, each hepatic schizont releases thousands of merozoites into the bloodstream.

Eighty-five putative ePKs were found in the genome of *P. falciparum* 3D7 (Ward et al., 2004), but only a small fraction of these have so far been assigned functions. Representatives of 5 of the 7 major ePK subgroups were recognized, as were a large number of unusual ePKs, dubbed orphans, which include a unique family characterized by a conserved phenylalanine-isoleucine-lysine-lysine (FIKK) amino acid motif in subdomain II.

Protein kinases have become important targets in the fight against cancer and other diseases (Zhang et al., 2009), and it has been argued that many protein kinases of *Plasmodium* have diverged sufficiently from those of their human hosts to offer targets for selective inhibitors to combat malaria (Doerig and Meijer, 2007). A large proportion of the thousands of drug-like compounds that recent cellular screens identified as blocking *P. falciparum* invasion or growth in vitro (Gamo et al., 2010; Guiguemde et al., 2010; Plouffe et al., 2008) are ATP analogs. While reflecting in part the bias in industrial screening libraries, these data now also imply protein kinases as the largest group of putative targets for some of the most promising lead compounds. Progress toward the genetic validation of all *Plasmodium* protein kinases is therefore urgently required.

So far only three ePKs of *P. falciparum* were shown conclusively to be essential for asexual blood stage development (Dorin-Semblat et al., 2007; Dvorin et al., 2010; Holland et al., 2009). We propose that the genetically more tractable rodent parasite offers a unique opportunity for an urgently needed systematic and unbiased analysis of protein kinase functions throughout the malaria life cycle in vivo, including during mosquito transmission. We show that the kinome of *P. berghei* is highly conserved when compared to *P. falciparum.* In a global deletion analysis, we identify 23 ePK mutants that can complete asexual erythrocytic development of *P. berghei* in mice. Through phenotypic screening of all ePK mutants at four key life cycle stages in vitro and in mosquitoes, we identify clusters of functionally related genes that provide starting points for the in-depth analysis of sexual development and sporogony. We also illustrate how a comprehensive deletion analysis can guide target selection for in vitro drug screening against asexual blood stages by deprioritizing genes that are functionally redundant in vivo.

## Results and Discussion

### The Kinome Is Largely Conserved between *Plasmodium* Species

The domain signature of conventional eukaryotic protein kinases (ePKs) comprises 12 conserved subdomains, designated I–XII, which fold into a common catalytic core structure (Hanks and Hunter, 1995). In the *P. berghei* ANKA genome, we identified 73 gene models with putative protein kinase domains (Table S1). The predicted amino acid sequences of all ePK domains from *P. berghei* and *P. falciparum* were aligned to identify three invariant active site residues typically found in kinases that are catalytically active: an ATP-binding lysine in subdomain II, a conserved aspartic acid in the catalytic loop formed by subdomain VIb, and a conserved aspartic acid in the DFG motif of subdomain VII that chelates the activating Mg^2+^ ion. All three invariant residues are conserved in 86 *P. falciparum* 3D7 genes, but in only 66 putative *P. berghei* kinases (Figure S1A). Five divergent *P. berghei* sequences lack one or more key catalytic residues, although most ePK subdomains are recognizable. We classify these genes, which are all conserved in *P. falciparum*, as putative pseudokinases (Table S1). Two other unconventional protein kinases are predicted active members of the universally conserved RIO (*ri*ght *o*pen reading frame) family required for ribosome biogenesis.

**Figure 1:**
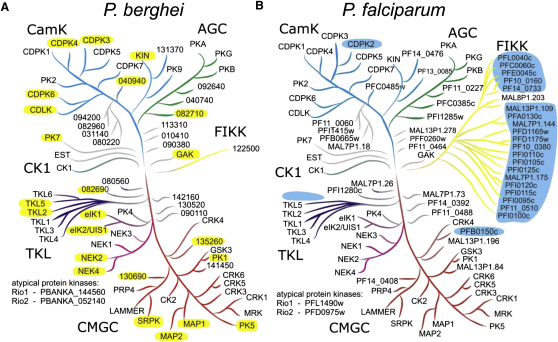
Phylogenetic Trees of *Plasmodium* ePK Domains (A) Protein kinases of *P. berghei* ANKA. Genes that were disrupted in this study are highlighted in yellow. Colored branches reflect associations with conserved ePK groups. Gray branches indicate orphan kinases and some conserved ePKs of uncertain association. Only the numeric part of systematic gene IDs is shown on the tree. Refer to Figure S1B for bootstrap values supporting individual nodes and see Table S1 for additional data. (B) Protein kinases of *P. falciparum* 3D7. Highlighted in blue are the features that distinguish this kinome from that of *P. berghei* (see also Table S2). Both panels are derived from the alignment and neighbor-joining tree shown in Figure S1.

To determine which parts of the kinome have diverged between *P. berghei* and *P. falciparum*, we performed a phylogenetic analysis of all their 151 ePK domains. Selected human ePK domains were included to mark major kinase subgroups. In the resulting neighbor-joining tree (Figure S1B), most branches were crowned by pairs of kinases from both *Plasmodium* species. Sixty-four orthologous pairs were defined in this way, each supported by a highly significant bootstrap value of 99%–100%. All orthologous gene pairs were also syntenic in *Plasmodium*. From this three-species analysis, we derived species-specific trees, which are compared in Figure 1. The topology of both trees is very similar and consistent with the previous analysis for *P. falciparum* (Ward et al., 2004), with most *P. berghei* ePK domains falling into five subgroups that are widely conserved in eukaryotes. A smaller number of ePKs, sometimes referred to as “orphans,” could not be assigned to existing groups. Both *Plasmodium* kinomes lack tyrosine kinases, which are thought to have evolved only in the lineage leading to choanoflagellates and metazoa (Manning et al., 2008), possibly from a gene of the tyrosine kinase-like (TKL) group that is well represented in malaria parasites. Our analysis also confirms the unique absence of members of the widely conserved STE group, named after sterility phenotypes in yeast, which in other eukaryotes have critical functions, i.e., as upstream activators of mitogen-activated protein (MAP) kinases, two homologs of which are present in *Plasmodium* (Dorin-Semblat et al., 2007).

Only four features distinguish the kinomes of *P. berghei* and *P. falciparum* (highlighted in blue in Figure 1B): (1) In *P. berghei*, the TKL group has a sixth member, *tkl6*. Orthologs of *tkl6* are present in all sequenced malaria genomes, with the exception of *P. falciparum* and its closest relative, *P. reichenowi*, which infects chimpanzees and gorillas (Table S2). *tkl6* is the only ePK gene of *P. berghei* without ortholog in *P. falciparum*, demonstrating the high degree of kinome conservation across malaria species. (2) *P. berghei* and related *Plasmodium* species of rodents lack orthologs of PFB0150c, the *Plasmodium* kinase that comes closest to the Ste group of ePKs. (3) All rodent and some other malaria genomes lack one of the Ca^2+^-dependent protein kinases, CDPK2. (4) In *P. falciparum*, the FIKK family of protein kinases is expanded to 21 members. *P. reichenowi* also has multiple *fikk* genes, but *P. berghei* and all other malaria genomes possess only one, which is a syntenic ortholog of MAL8P1.203. Together, these genes define the ancestral FIKK as two-exon gene with short N-terminal domain. Expansion of the FIKK family in *P. falciparum* and *P. reichenowi* follows acquisition of an N-terminal extension to the kinase domain encoded in part by a new 5′ exon, which provides critical functional signals for secretion and protein export to the host cell (Nunes et al., 2007).

To determine whether differences between the kinomes of *P. berghei* and *P. falciparum* are lineage-specific gene acquisitions or losses, we conducted a phylogenetic analysis using the bird malaria, *P. gallinaceum*, as outgroup (Martinsen et al., 2008). The incomplete *P. gallinaceum* genome contains putative orthologs of *tkl6*, PFB0150c, and *cdpk2* and has a single *fikk* gene, which is of the ancestral type (Table S2). This most likely reflects the situation in the last common ancestor of all *Plasmodium* species of mammals and shows that the evolution of *Plasmodium* kinomes is dominated by rare, lineage-specific gene losses. In marked contrast is the expansion of the FIKK group in the lineage leading to *P. falciparum* and *P. reichenowi*. Whether this expansion was driven by fitness benefits afforded by either functional or antigenic diversification remains to be investigated.

### Over a Third of *P. berghei* ePK Genes Are Redundant in Asexual Blood Stages

**Figure 2:**
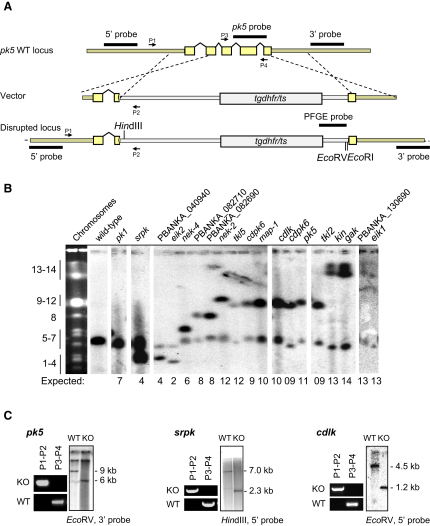
Strategy for Gene Targeting and Genotyping (A) The typical strategy for gene deletion is illustrated, using the *cdc2* homolog *pk5* as an example. Also shown are primers P1–4 used for diagnostic PCR analysis, probes used for Southern hybridization, and diagnostic restriction sites. Refer to Figure S2 for all vector designs and genotyping data. (B) Pulsed-field gel analysis of 18 mutants confirms integration of targeting vectors into the expected chromosome. Chromosome blots were hybridized with a probe recognizing the *tgdhfr/ts* resistance cassette, which also hybridizes more weakly to the *P. berghei dhfr* 3′ UTR on chromosome 7. Only the first clone obtained for each mutant is shown. (C) Diagnostic PCR (left) and Southern hybridization (right) with target-specific probes showing disruption of *pk5*, *srpk*, and *cdlk* genes.

To identify kinases that are redundant during the asexual erythrocytic phase of the life cycle, we next attempted to delete each of the 73 kinase-like genes in *P. berghei*. Most targeting vectors were designed to replace irreversibly the protein coding region with the *tgdhfr/ts* resistance marker by double homologous recombination. In Figure 2A, this is illustrated for protein kinase 5 (*pk5*), a homolog of mammalian *c*ell *d*ivision *c*ontrol kinase, *cdc2*. Where gaps in the known genome sequence imposed alternative strategies, we aimed to delete or disrupt at least the protein kinase domain (see Figure S2A for all construct designs). Insufficient sequence data prevented targeting of two ePKs (Table S1). Integration of targeting vectors into the predicted chromosome was confirmed for all mutant clones by hybridizing chromosome blots with a probe recognizing the resistance marker (Figure 2B). Disruption of the target gene was then confirmed by diagnostic PCR and Southern hybridization of digested genomic DNA (Figures 2C and S2B).

348 individual gene deletion attempts—at least three per gene—led to the disruption or deletion of 23 of 66 ePK genes (35.4%) (highlighted in yellow in Figure 1A). Most mutants were obtained as multiple clones from independent experiments. Only one cloned mutant displayed a mild growth phenotype during asexual development in mice (see below). For three additional ePK genes, *cdpk7*, *tkl3*, and *PBANKA_130520*, genotyping was reproducibly positive in a subpopulation of parasites after the initial round of drug selection, but dilution cloning failed, probably due to insufficient fitness of the mutants. As was expected for a haploid organism that requires extensive in vivo selection, we only managed to delete genes that were completely or largely redundant for asexual erythrocytic development in mice. We therefore screened all mutants for phenotypes during sexual development and transmission through *Anopheles* mosquitoes.

### Phenotype Analysis Reveals Kinase Functions in Sexual Development

**Figure 3:**
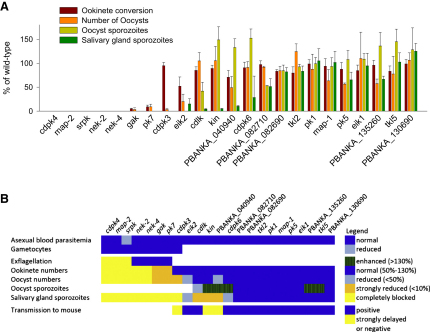
Phenotypic Screen of 23 ePK Mutants (A) Data from 43 transmission experiments are summarized. Mutants are arranged by life cycle stage and decreasing severity of the phenotype. Error bars show standard deviations from at least three independent experiments per mutant (also refer to Table S3). (B) Heat map representation of all phenotyping data. White space indicates the phenotype was not determined.

**Figure 4:**
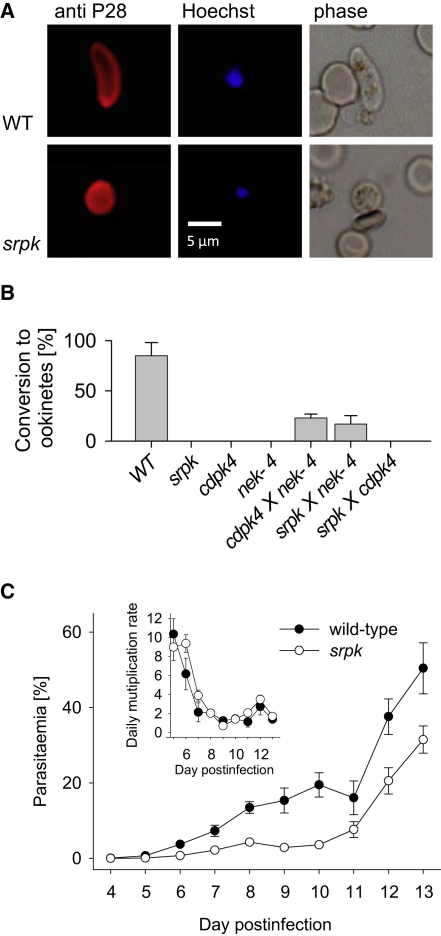
Characterization of an *srpk* Mutant (A) Fluorescence microscopy illustrating a P28-positive wild-type ookinete (upper row) and a P28-positive *srpk* macrogamete (lower row). The latter remained unfertilized, as indicated by weaker Hoechst labeling of nuclear DNA, consistent with the inability of the *srpk* mutant to produce microgametes. (B) Cross-fertilization experiments to examine the competence of *srpk* macrogametes to differentiate into ookinetes when fertilized by microgametes from a female defective mutant, such as *nek-4*. Crosses are not expected to restore ookinete conversion to wild-type levels, because around half of macrogametes in crosses are *nek-4^−^* and therefore blocked in differentiation. Error bars show standard deviations of three biological replicates. (C) Average daily parasitemia in six BALB/c mice infected with 1 × 10^3^ wild-type or *srpk* parasites. The inset shows daily multiplication rates calculated as (parasitemia_day n_/parasitemia_day n__− 1_). Error bars give standard errors of the mean. Growth curves are representative of three experiments with two independently generated knockout clones.

First, we screened for the ability of all mutants to complete sexual development and progress to the ookinete stage in vitro. When gametocyte-containing blood is cultured for 24 hr under conditions that trigger gamete formation, *P. berghei* efficiently forms stationary zygotes, which then differentiate into motile ookinetes. Using surface expression of the P28 antigen as a stage-specific marker, we quantified the proportion of round macrogametes and zygotes that developed into typically banana-shaped ookinetes (Figures 3 and 4A). For 16 mutants, macrogamete-to-ookinete conversion (Figure 3) and ookinete numbers (data not shown) were similar to wild-type. Since ookinete formation relies on the fusion of male and female gametes, we conclude that in these mutants, all aspects of sexual development were close to normal.

In contrast, seven mutants were affected strongly in their ability to produce ookinetes in vitro (Figure 3). Four of these, *cdpk4*, *map-2*, *nek-2*, and *nek-4*, were described previously. Among the mutants, a complete block of ookinete formation was caused by deletion of the putative *Plasmodium* homolog of serine/arginine-rich (SR) protein kinase (SRPK), a pre-mRNA splicing factor that is highly conserved in all eukaryotes. By phosphorylating the serine residues of arginine-serine dipeptides in RS domains of SR proteins, SRPKs promote both constitutive splicing of pre-mRNA and alternative splice site selection (Manley and Tacke, 1996).

In two other mutants, the developmental block remained incomplete, although ookinete formation was reduced by >90%. One carries a deletion of protein kinase 7 (*pk7*), a hybrid kinase with homology to mammalian MEK3/6 and fungal protein kinase A in the C- and N-terminal regions of its kinase domain, respectively. In *P. falciparum*, PK7 is important for mosquito transmission (Dorin-Semblat et al., 2008), and our data from *P. berghei* now define its critical function more precisely as being before ookinete formation. In *P. falciparum*, deletion of *pk7* also reduces the rate of intraerythrocytic replication as a result of fewer daughter merozoites forming per erythrocytic schizont. In *P. berghei* blood stages, we did not observe an obvious growth phenotype; however, we did not rigorously examine growth rate in coinfection experiments with wild-type. The third ookinete phenotype resulted from deletion of a putative ePK whose deduced amino acid sequence resembles that of the Ark/Prk group of yeast kinases and the cyclin G-associated kinase (GAK)/AP2-associated kinase 1 (AAK1) group in animals. Both function in uncoating clathrin-coated vesicles (Smythe and Ayscough, 2003).

### A Putative SRPK Is Required for Exflagellation In Vitro

*Srpk*, *pk7*, and *gak* mutants formed morphologically normal gametocytes (data not shown), and ookinete cultures contained macrogametes that had emerged from their host cells expressing the activation marker P28 (i.e., Figure 4A), suggesting that neither gametocytogenesis nor early events in macrogametocyte activation were responsible for the lack of ookinete production. We therefore asked if microgamete formation was affected in the mutants. Normal levels of exflagellation were observed in *pk7* and *gak* in vitro, but microgamete formation was blocked completely in *srpk* (Figure 3B). This prompted us to examine whether female *srpk* gametes were fully viable by asking whether they could convert to ookinetes when fertilized by microgametes from a female-defective *nek-4* null mutant (Reininger et al., 2005). *Nek-4* microgametes restored ookinete formation to similar levels when cocultured with either the *srpk* mutant or the *cdpk4* mutant (Figure 4B), which is known to have a male-specific exflagellation phenotype (Billker et al., 2004), confirming that *srpk* is essential specifically during male gamete formation. Alternative splicing through exon skipping and use of alternative splice acceptor sites occurs in multiple stages and across *Plasmodium* species and can be developmentally regulated (Singh et al., 2004). It is possible that the development of functional microgametocytes is particularly reliant on alternative splicing of an unidentified mRNA mediated by SRPK. However, SRPKs and SR proteins also contribute to cell-cycle progression through M phase (Zhong et al., 2009). Since microgametocytes progress through the cell cycle with extraordinary speed, producing eight gametes from a haploid cell within only 10 min, they may be particularly sensitive to the loss of a mitotic regulator.

*Srpk* also has a comparatively minor function during asexual erythrocytic development, since two independently created mutants both showed delayed growth in mice (Figure 4C). *Srpk* parasites were only mildly attenuated, and the peak replication rate during exponential growth was not reduced (Figure 4C, inset). Unlike in an attenuated *msp7* mutant (Tewari et al., 2005b), the *srpk* growth defect was also not due to enhanced reticulocyte restriction of the mutant. Redundancy within the SR kinase family of splicing factors may explain why *srpk*, like its ortholog *dsk1* in fission yeast (Bimbó et al., 2005), is largely dispensable in asexual erythrocytic stages. In *Plasmodium*, the SR kinase group includes an ePK of the clk/LAMMER type (Li et al., 2001) that we failed to delete. The likely ortholog of the essential yeast mRNA processing factor 4 (*prp4*) kinase also resisted knockout in *P. berghei.* In contrast, a more distantly *clk*-related gene, *PBANKA_130690*, could be deleted without apparent phenotype at any life cycle stage we investigated (Figure 3).

### *gak* and *pk7* Are Regulators of Zygote Development

Four mutants with normal gametogenesis, which then failed to progress to the ookinete stages in vitro, were *nek-2*, *nek-4*, *pk7*, and *gak* (Figure 3). *Nek-2* and *nek-4* encode kinases of the NIMA (*n*ever *i*n *m*itosis gene *a*) family involved in controlling centrosomes and entry into mitosis. Both are catalytically active kinases that are introduced into the zygote by the female gamete and required at or before the genome replication step that precedes meiosis (Reininger et al., 2005, 2009). Two other NIMA family members, *nek-1* and *nek-3*, could not be deleted. Since Nek-1 was previously characterized as specific to the male gametocyte proteome (Khan et al., 2005), its likely essential nature was unexpected. However, a mutation in *Toxoplasma gondii nek-1* leads to defects in the spindle apparatus and causes chromosome missegregation in asexually replicating tachyzoites (Gubbels et al., 2008). Nek-1 may thus be a more general regulator of the apicomplexan cell cycle, which is only upregulated in microgametocytes due to the rapid completion of multiple cell cycles at this life cycle stage.

The exact points at which *pk7* and *gak* mutants are blocked in ookinete formation remains to be investigated. *Plasmodium* GAK is the best candidate in the kinome to regulate clathrin-mediated events (Shimizu et al., 2009). The *gak* mutant may provide a starting point to study the unexplored functions of clathrin-mediated vesicle trafficking, i.e., for protein targeting to secretory organelles or possibly during formation of the inner membrane complex, which are both key to the parasitic life style of *Plasmodium*.

Unexpectedly, no mutants defective in gametocyte formation were recovered. This could mean the switch to sexual development does not rely on its own kinase pathway or that gametocytogenesis is the default route for intraerythrocytic parasite differentiation, in which case disruption of a regulatory pathway diverting all parasites into asexual development would appear lethal.

### 12 ePKs Are Required for Parasite Transmission to the Mosquito In Vivo

To identify phenotypes affecting in vivo transmission, we screened all 23 viable ePK mutants by allowing groups of mosquitoes to feed on mice carrying gametocytes. At different times postinfection (p.i.), mosquitoes from each batch were removed and examined for oocysts on the midgut (day 10 p.i.) and for sporozoites associated with the midgut (day 14 p.i.) or the salivary glands (day 21 p.i.). All mutants blocked completely in gametogenesis or ookinete formation in vitro also failed to transmit and reach the oocyst stage in vivo (Figure 3 and Table S3), demonstrating a good correlation between experiments. The only mutant that formed ookinetes in vitro but failed to produce oocysts in vivo was the previously described *cdpk3* mutant (Figure 3), which is defective in gliding motility and therefore strongly compromised in its ability to penetrate the midgut epithelium (Siden-Kiamos et al., 2006). No other mutants with a comparable phenotype were recovered, presumably because other protein kinases involved in regulating gliding and midgut invasion, such as CDPK1, PKB, and PKG, are most likely essential in the merozoite and could not be deleted. In total, 12 ePK mutants were either completely or partially compromised in their ability to reach the mosquito salivary glands (Figure 3).

### *gak*, *pk7*, and a Putative CDPK-like Kinase Have Different Roles in Sporogony

**Figure 5:**
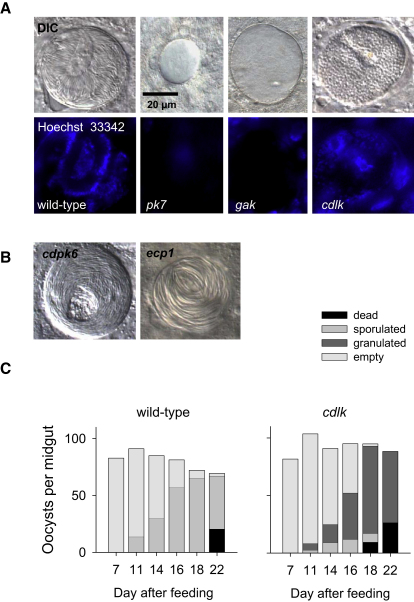
Oocyst Phenotypes (A) Representative images of wild-type and mutant oocysts on mosquito midguts dissected 21 days after feeding. (B) A representative *cdpk6* oocyst compared to a previously characterized *ecp1* cysteine protease mutant with egress phenotype. (C) Quantitative analysis of the oocyst granulation in a *cdlk* mutant. At each time point, 5–10 wild-type and *cdlk* infected midguts were dissected and labeled with SYTOX green, and oocysts were assigned to categories. Oocysts showing signs of sporoblast formation or sporozoites were counted as sporulated. Oocysts labeled with SYTOX green were scored as dead.

An ookinete that has penetrated the midgut epithelium stops gliding and rounds up. The apical complex is reabsorbed into the cytoplasm and the cell differentiates into an oocyst. Days of cellular growth are accompanied by sustained mitotic activity within an intact nuclear envelope until the cell eventually reorganizes into sporoblast regions, each forming a budding center for hundreds of sporozoites (Aly et al., 2009). Although the oocyst is the longest replication phase in the malaria life cycle, it remains the least well understood. Two mutants with early phenotypes in sporogony are *gak* and *pk7*. Both produced <10% of wild-type oocysts, reflecting the lower rate at which ookinetes were generated by these mutants. Presumably, the ookinetes that did form left the midgut lumen effectively, since enough *gak* and *pk7* oocysts were present to analyze their fate further. By day 10 p.i., oocysts of neither mutant had formed sporoblasts, and no sporozoites were later found in infected midguts or salivary glands (Figure 3 and Table S3). *pk7* oocysts stopped growing around day 10 p.i., became susceptible to the dead cell marker SYTOX green, and had almost completely deteriorated by day 21 p.i. (data not shown). In marked contrast, *gak* oocysts continued to exclude SYTOX green and by day 21 had grown to an unusually large size (Figure 5A), while still not undergoing sporogony. Neither mutant gave rise to salivary gland sporozoites. Different oocyst phenotypes indicate *gak* and *pk7* function in different pathways before nuclear replication and sporoblast formation. Lack of replication makes them distinct from sporogony phenotypes of mutants lacking either circumsporozoite protein (Ménard et al., 1997) or *lap* genes (Raine et al., 2007).

A striking phenotype late during sporogony was generated by deletion of *PBANKA_101980*, a gene encoding a previously unstudied member of the CaMK group with an ePK domain most similar to the CDPKs but that lacks the calmodulin-like domain typical of this family. This kinase, conserved across the apicomplexa, does not belong to the plant family of CDPK-related kinases (CRKs) with degenerate EF hands (Harper and Harmon, 2005), and we refer to it as *CD*PK-*l*ike *k*inase (CDLK) to reflect its independent evolutionary origin. Two independently produced *cdlk* deletion clones produced normal numbers of oocysts, which gave rise to a reduced number of sporozoites, such that the parasite load of salivary glands was reduced >95% on day 21 p.i. (Figure 3). *Cdlk* oocysts showed apparently normal DNA replication and by day 10–12 p.i. had initiated sporoblast formation. At the same time, we noted the appearance of oocysts with unusually granular content (Figure 5A). A quantitative time course analysis demonstrated that *cdlk* oocysts became granulated at the same rate at which wild-type oocysts underwent sporogony, suggesting CDLK could have an important function during sporozoite morphogenesis. Granulation was not associated with oocyst death, since abnormal oocysts persisted until day 21, and dying, SYTOX-positive oocysts appeared at the same rate in *cdlk* and wild-type infected mosquitoes (Figure 5C). Future work has to clarify the precise function for CDLK in processes such as cytoskeletal organization or inner membrane complex formation during sporogony.

### Three ePKs Are Required for Sporozoites to Reach the Salivary Glands

Three mutants with normal oocyst maturation showed a marked reduction of salivary gland infections (Figure 3). This could be due to a reduced ability of mutant sporozoites to resist immune attack in the mosquito hemolymph or a failure to recognize, invade, or survive within salivary glands. On the other hand, a concomitant increase in midgut-associated sporozoite numbers in all three mutants points toward their enhanced or prolonged retention within mature midgut oocysts and therefore to a defect in exit from the cyst. Egress is an active process, relying on a parasite cysteine protease, ECP1/SERA8 (Aly and Matuschewski, 2005). One mutant in this functional group carried a deletion of *cdpk6*, a gene encoding an unusual CDPK with a large N-terminal extension and atypically arranged EF hands for Ca^2+^ binding. Lending support to a reduced (or delayed) egress hypothesis, mature *cdpk6* oocysts often contained many trapped sporozoites in an unusual circular arrangement, which were sometimes moving and were similar in appearance to the *ecp1* egress mutant (Figure 5D). Unlike *ecp1*, *cdpk6* sporozoites reached the salivary glands in numbers sufficient for their further functional characterization (Coppi et al., 2007), which found them to have normal gliding motility in vitro, but a reduced ability to productively invade into a parasitophorous vacuole. A role for *cdpk6* in regulated secretion of parasite factors such as *ecp1* might reconcile both phenotypes. This would most likely involve Ca^2+^ as a second messenger interacting with the Ca^2+^-sensing domains of CDPK6.

Two other CaMK members with reduced salivary gland infections are *PBANKA_040940* and *kin* (Table S3). The former is a putative orphan kinase that bears little resemblance to ePKs outside the apicomplexa. Consistent with a previous report (Purcell et al., 2010), salivary gland sporozoites of this mutant were reduced by more than 90%, but sufficient sporozoites remained to expect these to transmit back to mice if fully infectious. However, three attempts to transmit this mutant by allowing 5–15 infected mosquitoes to feed on highly susceptible C57BL/6 mice failed, suggesting *PBANKA_040940* may have a second function after salivary gland invasion.

*kin* is a *Plasmodium* homolog of sucrose nonfermenting (Snf) 1-related/AMP-activated kinases. From yeast to mammals, Snf1/AMP kinases are characterized by a ubiquitin-associated domain immediately following the kinase domain (Panneerselvam et al., 2006), which we find is conserved in *Plasmodium* KIN, although its detection required a structure-based approach to find remote homology (Kelley and Sternberg, 2009). Snf1/AMP kinase is a key regulator of cellular energy metabolism and is also involved in metabolite sensing and stress responses (von Plehwe et al., 2009). The redundancy of *kin* in intraerythrocytic stages is worth noting, since the only eukaryote lacking a Snf1 ortholog is the obligate intracellular parasite *Encephalitozoon cuniculi* (Miranda-Saavedra et al., 2007). The life style of intracellular parasites may obviate the need to keep energy metabolism tightly controlled. *kin* may therefore only be required to control metabolism and stress responses in sporozoites, which are extracellular and most likely exposed to varying nutrient levels in the mosquito and which presumably require a large increase in metabolic activity for gliding upon entering the vertebrate host while at the same time coping with a temperature shock. We were able to transmit *kin* knockout sporozoites back to C57BL/6 mice by mosquito bite, but only with a much-extended prepatent period of ∼15 days, a very significant delay that is not easily explained by the 95% reduction in salivary gland-associated sporozoites in the same batch of infected mosquitoes.

**Figure 6:**
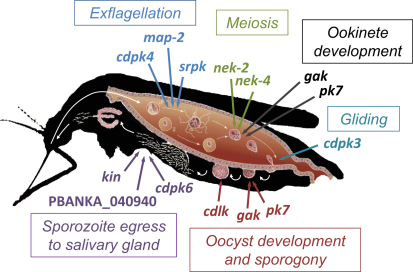
Schematic Illustration of Stage-Specific Kinase Gene Functions in *P. berghei* Mosquito Stages *Cdpk4*, *map-2*, and *srpk* are essential for male gamete formation. *Nek-2* and *nek-4* are individually essential for DNA replication that precedes meiosis in the zygote. In *gak* and *pk7* mutants, zygotes largely fail to differentiate into ookinetes; a small number of oocysts are produced but fail to sporulate. *Cdpk3* knockout ookinetes are defective in gliding motility and invasion of the mosquito midgut epithelium. *Cdlk* mutant oocysts fail late during development, at about the stage of sporoblast formation. Oocysts of parasites lacking *kin*, *cdpk6*, or *PBANKA_040940* have fewer sporozoites in the salivary glands, which may be linked to their enhanced retention in the oocyst. Figure modified from Billingsley and Sinden (1997).

Figure 6 summarizes the conclusions from our phenotypic screen. Of 23 ePK mutants, only 8 were blocked completely before salivary gland invasion. Of the remaining 15 mutants, three showed a reduction in salivary gland sporozoites to below 10% of wild-type, and all but one were transmitted back to mice by mosquito bite. Ten other ePK mutants lacked a clear phenotype. Importantly, a more quantitative analysis of sporozoite infectivity than was possible here may yet reveal additional phenotypes at the liver stage in these mutants.

### No More Than 43 ePKs Constitute the Essential Kinome of Asexual Erythrocytic Stages

Failure to disrupt a gene provides insufficient evidence for its essentiality. We therefore refer to such genes as “possibly essential.” Genes may be wrongly attributed to this category, i.e., because gene targeting in *P. berghei* is not completely effective. In fact, in this study, only 75.3% of individual targeting attempts of redundant genes led to deletion of the target. A maximum-likelihood model estimates the number of essential genes that provide the best statistical fit, considering the outcome of all 348 deletion attempts, as 42.9, close to the 43 we observed. However, the model assumes an equal probability of deleting each redundant gene. In reality, targeting frequencies in eukaryotes can vary widely with chromosomal location (Hasty et al., 1994; Papadopoulou and Dumas, 1997). Furthermore, most loss-of-function mutations have an intermediate impact on competitive fitness of an organism that depends on environmental conditions and genetic context (D'Elia et al., 2009). Therefore, direct genetic evidence is critical for claims of essentiality, and the development of suitable conditional genetic technologies in *Plasmodium* that can be scaled up to validate large numbers of drug targets systematically remains a priority. With these caveats in mind, the successful deletion of 23 ePK genes nevertheless provides an upper limit estimate of 43 for the essential *P. berghei* kinome (including two ePKs we did not study).

### Drug Target Prioritization

With the core kinome largely conserved between *Plasmodium* species, showing that over a third of ePK genes are nonessential for *P. berghei* asexual erythrocytic stages in vivo provides an effective way of deprioritizing a large part of the kinome as targets for drugs directed against this life cycle stage. Importantly, many genes that in *P. falciparum* have been pursued for drug development are not among the redundant group. Examples of current blood stage targets that we failed to delete include *mrk* (the *cdk7* homolog), *cdpk1*, *gsk3*, *ck1*, *pkg* (which is important in *P. falciparum* schizogony) (Taylor et al., 2010), and *crk1*, confirming a previous study by Rangarajan et al. (2006) showing the gene is essential. On the other hand, we unexpectedly deleted *pk5* (Figure 2), the *Plasmodium* homolog of *cdc2*, without obvious phenotype, although this kinase is an essential regulator of the cell cycle in yeast (Holton et al., 2003). This highlights not only the difficulty of predicting gene functions reliably, but also the need for experimental target validation.

A major challenge for an antimalarial kinase inhibitor will be to achieve selectivity over human protein kinases while ideally blocking multiple essential parasite enzymes to prevent the rapid emergence of drug resistance. One way to achieve this would be by focusing efforts on multiple essential targets within a unique subgroup of parasite kinases, such as the CDPKs, which have a domain architecture and mode of regulation otherwise found only in plants. Within this family, recent efforts to inhibit CDPK1 (Kato et al., 2008) could be complemented by CDPK5 as additional target, consistent with its recently confirmed function in *P. falciparum* schizont rupture (Dvorin et al., 2010). CDPK4 is a useful secondary target for a pan-CDPK inhibitor, because of its absolute and early requirement for transmission. Other CDPK family members and CDPK-related CaMKs can be deprioritized because they are either not essential in at least one *Plasmodium* species or, in the case of CDPK2, absent from most malaria species infecting humans.

Clinically important inhibitors of human oncogenic tyrosine protein kinases achieve relative selectivity for their targets by interacting with a hydrophobic pocket at the back of the ATP binding site, access to which is blocked in most ePKs by a large “gatekeeper” residue. Where such a gatekeeper is small, it can be exploited as a selectivity filter for rational inhibitor design (Cohen et al., 2005). This has been demonstrated for a class of anticoccidial inhibitors of *Toxoplasma* and *Plasmodium* PKG, which achieve selectively over the human ortholog by exploiting the small threonine gatekeeper found only in the coccidial enzyme (Donald et al., 2002; McRobert et al., 2008), and more recently for *T. gondii* CDPK1 (Ojo et al., 2010).

**Figure 7:**
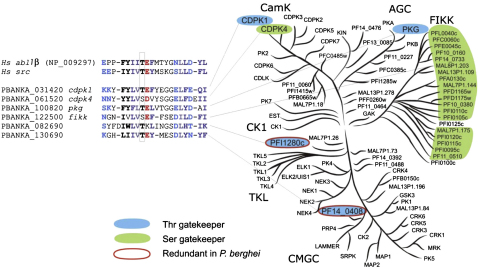
Target Prioritization among Small Gatekeeper Kinases of *Plasmodium* Subdomain V of all six *P. berghei* ePKs with small gatekeeper is aligned with the human tyrosine kinase Src and the cancer target Abl. *P. falciparum* homologs with small gatekeepers are shown on the tree, and redundancy in *P. berghei* is highlighted in red.

Gatekeeper residues predicted from the kinase domain alignment (Figure S1A) are perfectly conserved between *P. falciparum* and *P. berghei* (Table S1). In the conserved core kinome, six *Plasmodium* ePKs have a small (threonine) or very small (serine) gatekeeper residue (Figure 7A) that may facilitate the design of selective inhibitors. In addition to the likely essential targets PKG, CDPK1, and the ancestral FIKK kinase (Figure 7B), these include CDPK4. Two uncharacterized putative kinases with small gatekeepers, however, can be deprioritized, because we could delete both without apparent phenotype. All but two members of the expanded family of likely exported FIKKs in *P. falciparum* 3D7 share small serine gatekeepers, providing a rationale for targeting almost the entire family (data not shown).

## Experimental Procedures

### Sequence Analysis Methods

*P. berghei* protein kinases and ePK domain boundaries in the May 2010 assembly of the *P. berghei* ANKA genome (ftp://ftp.sanger.ac.uk/pub/pathogens/P_berghei/May_2010_assembly/) were identified using Hidden Markov Model (HMM) profiles for protein kinase domains (Finn et al., 2010). Additionally a six-frame translation of the *P. berghei* ANKA 8× genome assembly was searched for local alignments (Altschul et al., 1997), with 85 protein kinase domains identified previously in *P. falciparum* 3D7 (Ward et al., 2004).

### Generation and Genotyping of Gene Knockout Parasites

DNA vectors to replace target genes by double homologous recombination were generated by inserting 0.5–1.0 kb of 5′ homologous sequence and 0.4–1.0 kb of 3′ homologous sequence on either side of a *T. gondii dhfr/ts* expression cassette that confers resistance to pyrimethamine. Linear targeting vectors were released from the cloning plasmid by restriction digest, and *P. berghei* ANKA strain 2.34 was then transfected by electroporation (Janse et al., 2006). For initial genotyping of recombinant parasite pools, chromosome blots were hybridized with a probe recognizing the *pbdhfr/ts* 3′ UTR, which is present in the resistance cassette. Following dilution cloning of mutants, deletion of the targeted region of the genome was verified by PCR and Southern blot analysis. See Table S5 for all oligonucleotide sequences used in this study.

### Phenotype Screening of Mutants

Asexual growth rates were determined in groups of six BALB/c mice injected intravenously with 1000 parasites. Parasite growth was monitored on Giemsa-stained thin blood films, counting parasites daily in 1,000–40,000 erythrocytes until mice developed severe disease.

To screen mutants for their ability to undergo sexual development and sporogony, groups of 3–4 outbred Theiler's Original mice were infected with 5 × 10^6^ mutant or wild-type parasites intraperitoneally and monitored for gametocyte production on Giemsa-stained thin blood films. On day 3–4 p.i., blood samples from each mouse were taken from the tail and mixed with gametocyte-activating medium. Short-term cultures were set up to quantify exflagellation of microgametocytes (after 10 min by phase contrast microscopy) and macrogamete-to-ookinete conversion (at 24 hr after fluorescence labeling of the activation marker P28). Each mouse was then anesthetized, and ∼100 female *Anopheles stephensi* mosquitoes were allowed to feed for 20 min. Unfed mosquitoes were removed the following day. Batches of infected mosquitoes were dissected at different time points after feeding to monitor parasite development. On day 10, around 20 midguts were individually inspected by phase contrast microscopy, and oocysts were counted. On day 14, around 20 midguts were homogenized gently to release sporozoites, which were then counted in a hemocytometer and expressed as average per mosquito. On day 21 after feeding, this procedure was repeated with salivary glands. After 21 days, any remaining infected mosquitoes were allowed to feed on 3 or 4 naive C57BL/6 mice in groups of 5–10. These mice, which are highly susceptible to sporozoite challenge, were then monitored daily from day 4 to day 15 after feeding for blood infections to determine if mutants could complete the life cycle. See Supplemental Experimental Procedures for details of phenotyping assays.

## Supplemental Information

Document S1. Supplemental Experimental Procedures, Supplemental References, Two Figures, and Four Tables

Table S5. Oligonucleotides Used in This Study
