# Supplementary material for: The Systematic Functional Analysis of Plasmodium Protein Kinases Identifies Essential Regulators of Mosquito Transmission
Source: Cell Host Microbe. 2010 Oct 21;8(4):377–87. doi: 10.1016/j.chom.2010.09.006 (PMC2977076; doi:10.1016/j.chom.2010.09.006)
Supplement: Document S1. Supplemental Experimental Procedures, Supplemental References, Two Figures, and Four Tables [file mmc1.pdf]

**Supplemental Information**
Cell Host & Microbe, *Volume 8*

# The Systematic Functional Analysis of *Plasmodium* Protein Kinases Identifies Essential Regulators of Mosquito Transmission

Rita Tewari, Ursula Straschil, Alex Bateman, Ulrike Böhme, Inna Cherevach, Peng Gong, Arnab Pain, and Oliver Billker

## SUPPLEMENTAL EXPERIMENTAL PROCEDURES

### Generation of Gene Knockout Parasites

Oligonucleotides used to amplify targeting sequences from *P. berghei* genomic DNA are described in “Table oligonucleotides”. Depending on construct design, restriction digests with KpnI/SacII or ApaI/XbaI were used to release the linear targeting vector from the cloning plasmid. Electroporated parasites were mixed immediately with 100 µl of reticulocyte-rich blood from a phenylhydrazine treated, naive mouse and incubated at 37°C for 15 minutes. Re-invaded parasites were injected intraperitoneally into naive mice. From day 1 post infection pyrimethamine was applied in the drinking water for four days. Mice were monitored for 15 days to allow any slow growing mutants to reach patency. Drug selection was repeated after passage to a second mouse, and resistant parasites were used for cloning by limiting dilution, and genotyping.

### Genotype Analysis of Mutants.

Chromosomes of wild type and knockout parasites were separated by pulsed field gel electrophoresis (PFGE) on an LKB 2015 Pulsaphor system or CHEF DR III using a linear ramp of 60–500 s for 72 hr at 4V/cm. Gels were blotted and hybridized with a probe recognizing both the resistance cassette in the targeting vector and, more weakly, the 3’UTR of the *P. berghei dhfr/ts* locus on chromosome 7. Two diagnostic PCR reactions were used as illustrated in Fig. 2A. In one reaction primers 1 and 2 were used to determine correct integration of the selectable marker at the target locus. Primers 3 and 4 (Fig. 2A) verified deletion of the target gene. Sequences for the locus-specific primers (P1, P3, and P4) are given in Table S4. The generic primer P2 was either ol248 (5’-GATGTGTTATGTGATTAATTCATACAC-3’) or ol539 (5’-CAATGATTCATAAATAGTTGGACTTG-3’), depending on whether the 5’ or 3’ end of the *tgdhftr/ts* cassette was used to verify integration. Having confirmed integration on one end by diagnostic PCR, we usually used Southern blot analysis to probe integration on the opposite end of the construct. Genomic DNA from wild type and mutant parasites was digested with diagnostic restriction enzymes cutting once within the knockout cassette, as illustrated in Fig. 2A.The fragments were separated on a 0.8% agarose gel, blotted onto a nylon membrane,

and probed with a PCR fragment homologous to the *P. berghei* genomic DNA just outside of the targeted region.

**Phenotype Analysis**

Infections for phenotype screens were initiated by the intraperitoneal injection of infected blood containing $5x10^6$ parasites into mice that had been pretreated with 0.2 ml of 6 mg/ml phenylhydrazine in PBS intraperitoneally to induce reticulocytosis. Asexual stages and gametocyte production were monitored on Giemsa-stained blood films.

Exflagellation was examined on days 3-4 post infection. 5 µl of gametocyte-infected blood were obtained from the tail with a heparinized pipette tip and mixed immediately with 40 µl of ookinete culture medium (RPMI1640 containing 25 mM HEPES, 25% fetal bovine serum, 10 mM sodium bicarbonate, 50 µM xanthurenic acid at pH 7.6). The mixture was placed under a Vaseline-coated cover slip and 10-20 min later exflagellation centers were counted by phase contrast microscopy in 12-15 fields of view using a 63x objective and 10x ocular lens.

Ookinete formation was assessed on the same day. 10 µl of infected tail blood were obtained as above, mixed immediately with 40 µl ookinete culture medium, and incubated for 1 h at 19°C to allow completion of gametogenesis and fertilisation. Each culture was then diluted with 0.4 ml of ookinete medium and transferred to a 24-well plate, which was incubated at 20°C for a further 21–24 h to allow ookinete differentiation. Cultures were pelleted for 2 min at 5000 rpm and then incubating for 5min on ice with 50 µl of ookinete medium containing the DNA dye Hoechst 33342 to a final concentration of 5 µg/ml and a Cy3-conjugated mouse monoclonal antibody 13.1, which recognizes the P28 protein on the surface of ookinetes and any undifferentiated macrogametes or zygotes. Labeled cultures were placed on a microscope slide under a Vaseline-rimmed cover slip and P28-positive cells counted with a Leica DMR fluorescence microscope. Ookinete conversion was expressed as the percentage of P28 positive parasites that had differentiated into ookinetes. *In vitro* cross fertilisation experiments with the *srpk* mutant were carried out by mixing 5 µl volumes of tail blood containing equal number of either *srpk*, *cdpk4* or *nek-4* mutant gametocytes (Liu et al., 2008). Ookinete conversion was then determined as above.

For mosquito transmission experiments 50-100 *Anopheles stephensi* SD500 mosquito were allowed to feed for 20-30 min on anaesthetized mice that had been infected with wild type or mutant parasites 4-5 days earlier, had reached an asexual parasitaemia ~5-7% and were carrying comparable numbers of gametocytes as determined on Giemsa stained blood films. Unfed mosquitoes were removed the following day. On day 14 post feeding approximately 20 mosquitoes were dissected and oocysts on their midguts counted. DNA replication in the cysts was examined by staining with Hoechst 33342 for 10-15

minutes before guts were washed and mounted under Vaseline-rimmed cover slips. Images were recorded using a 63x oil immersion objective on a Leica DMR fluorescence microscope equipped with a Leica DC500 digital camera. On day 21 post feeding another 20 mosquitoes were dissected and their guts and salivary glands crushed separately in a loosely fitting homogenizer to release sporozoites, which were then quantified using a haemocytometer.

Due to day-to-day variations in transmission levels, all data were normalized to a matching number of wild type controls analyzed on the same day. Average total infection levels for wild type were as follows: 67.5 ± 7.0 % of female gametes transformed into ookinetes within 24 h *in vitro* (n = 25 cultures); 97.8 ± 55.4 wt oocysts were counted per infected mosquito midgut on day 14 p.i. (n = 43 experiments); 31,000 ± 7,400 sporozoites were obtained per mosquito midgut on day 21 p.i. (n = 34 experiments); 6,600 ± 2,800 sporozoites were recovered from salivary gland per mosquito on day 21 p.i. (n = 37 experiments).

All mutants with salivary gland infections were analysed for the infectivity of their sporozoites. 30-50 infected mosquitoes were allowed to feed for 20 min on 3 anesthetized naïve C57Bl/6 mice, which are highly susceptible to sporozoite challenge. Mice were monitored for blood stage infections from day 3 and until about day 15 post feeding.

## Supplemental References

Edgar, R.C. (2004). MUSCLE: a multiple sequence alignment method with reduced time and space complexity. BMC Bioinformatics *5*, 113.

Felsenstein, J. (1985). Confidence limits on phylogenies: An approach using the bootstrap. Evolution *39*, 783-791.

Goodstadt, L., and Ponting, C.P. (2001). CHROMA: consensus-based colouring of multiple alignments for publication. Bioinformatics *17*, 845-846.

Liu, Y., Tewari, R., Ning, J., Blagborough, A.M., Garbom, S., Pei, J., Grishin, N.V., Steele, R.E., Sinden, R.E., Snell, W.J., *et al.* (2008). The conserved plant sterility gene HAP2 functions after attachment of fusogenic membranes in Chlamydomonas and Plasmodium gametes. Genes Dev *22*, 1051-1068.

Saitou, N., and Nei, M. (1987). The neighbor-joining method: a new method for reconstructing phylogenetic trees. Mol Biol Evol *4*, 406-425.

Tamura, K., Dudley, J., Nei, M., and Kumar, S. (2007). MEGA4: Molecular Evolutionary Genetics Analysis (MEGA) software version 4.0. Mol Biol Evol *24*, 1596-1599.

Zuckerkandl, E., and Pauling, L. (1965). Evolutionary divergence and convergence in proteins. In Evolving Genes and Proteins, V. Bryson, and H.J. Vogel, eds. (New York, Academic Press), pp. 97-166.

# Tewari et al., Figure S1 A (top)

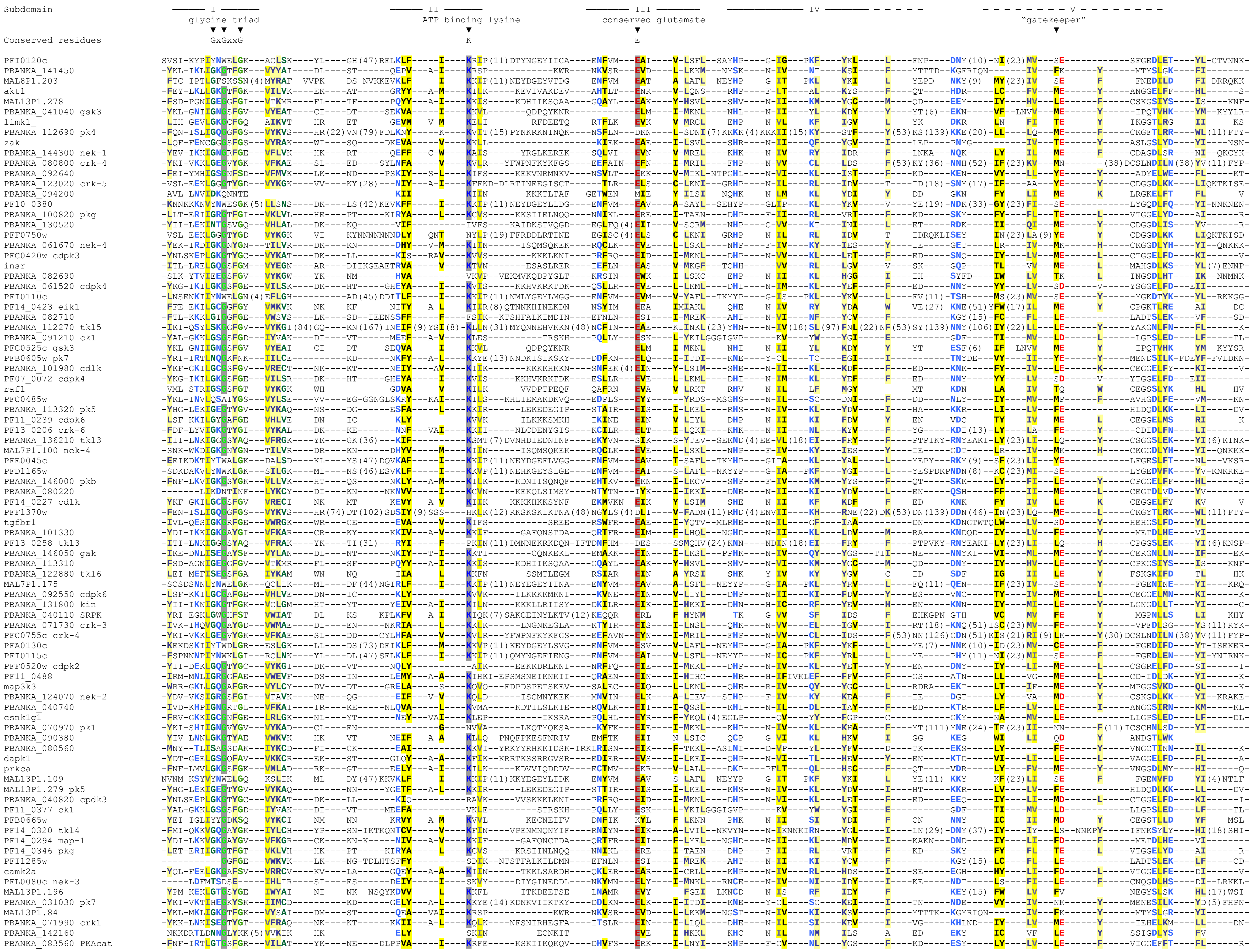

# Tewari et al., Figure S1 A (cont.)

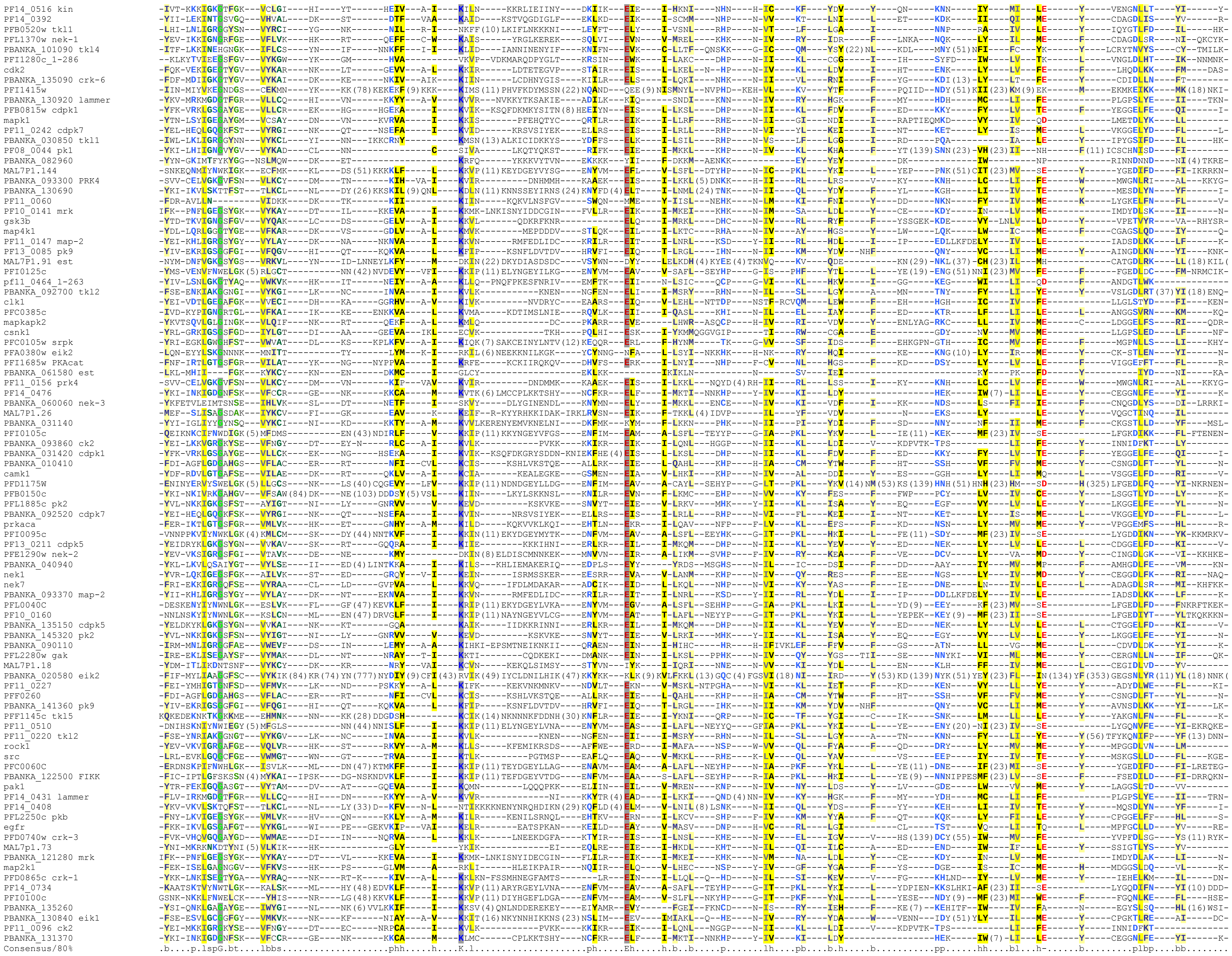

Tewari et al., Figure S1 A (cont.)

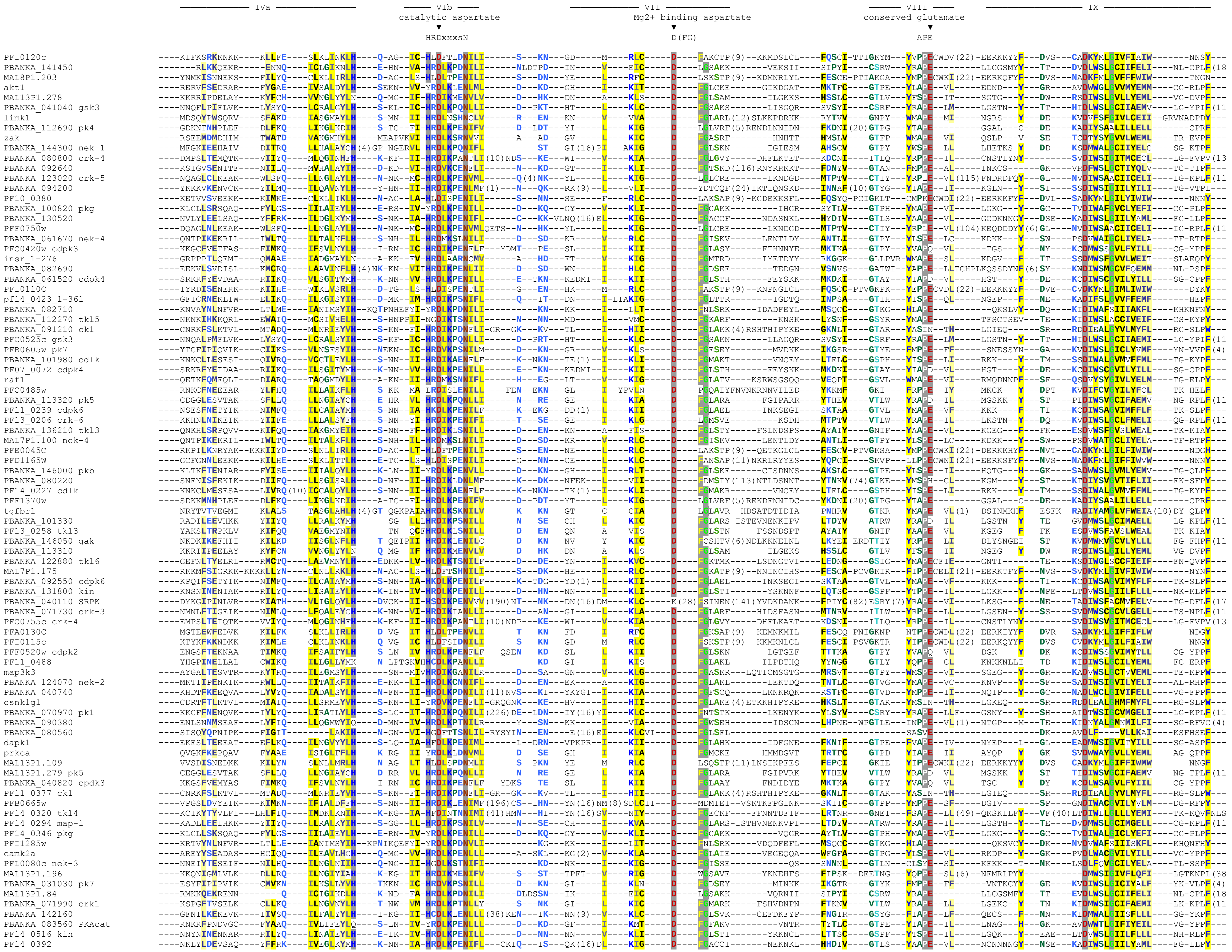

Tewari et al., Figure S1 A (cont.)

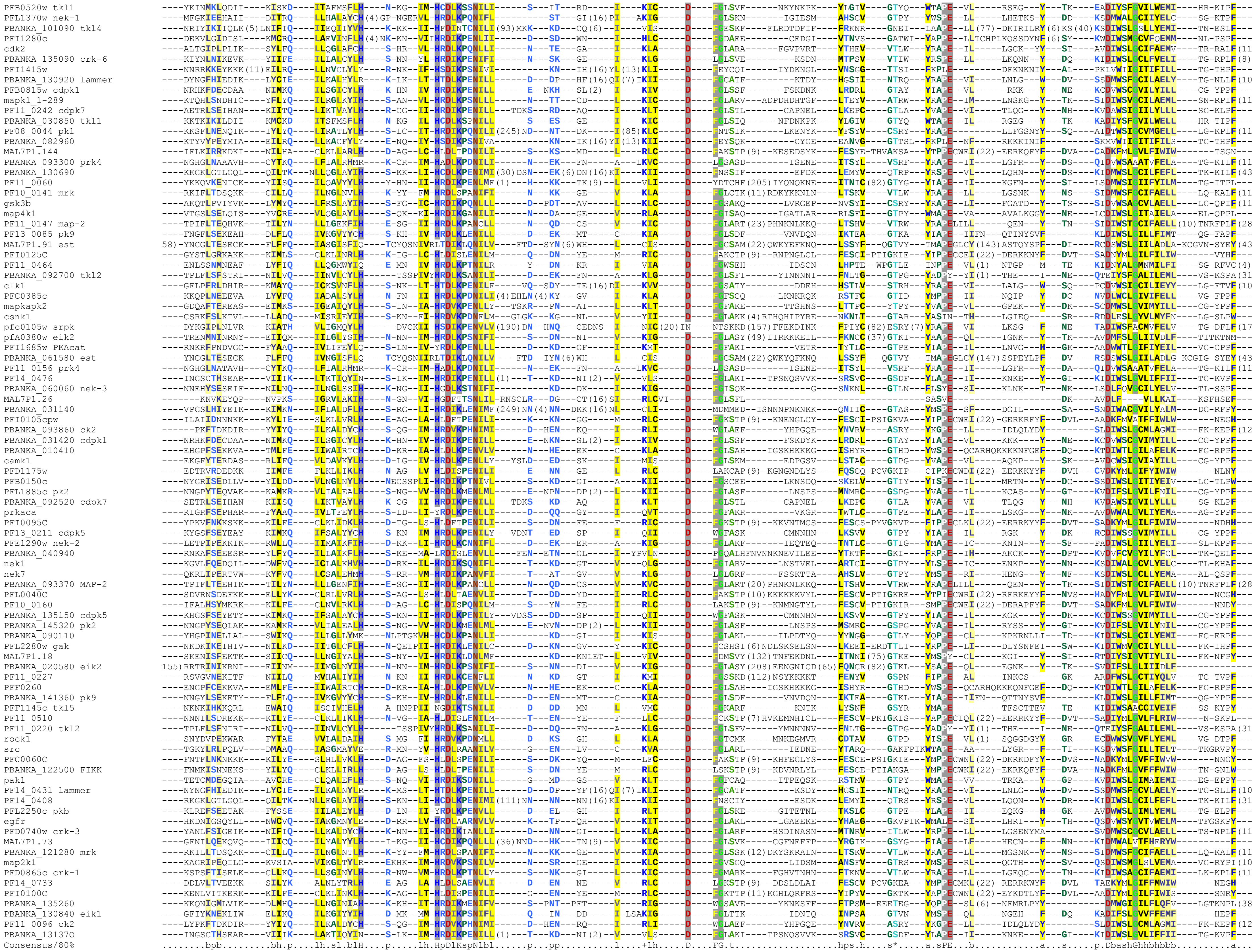

Tewari et al., Figure S1 A (cont.)

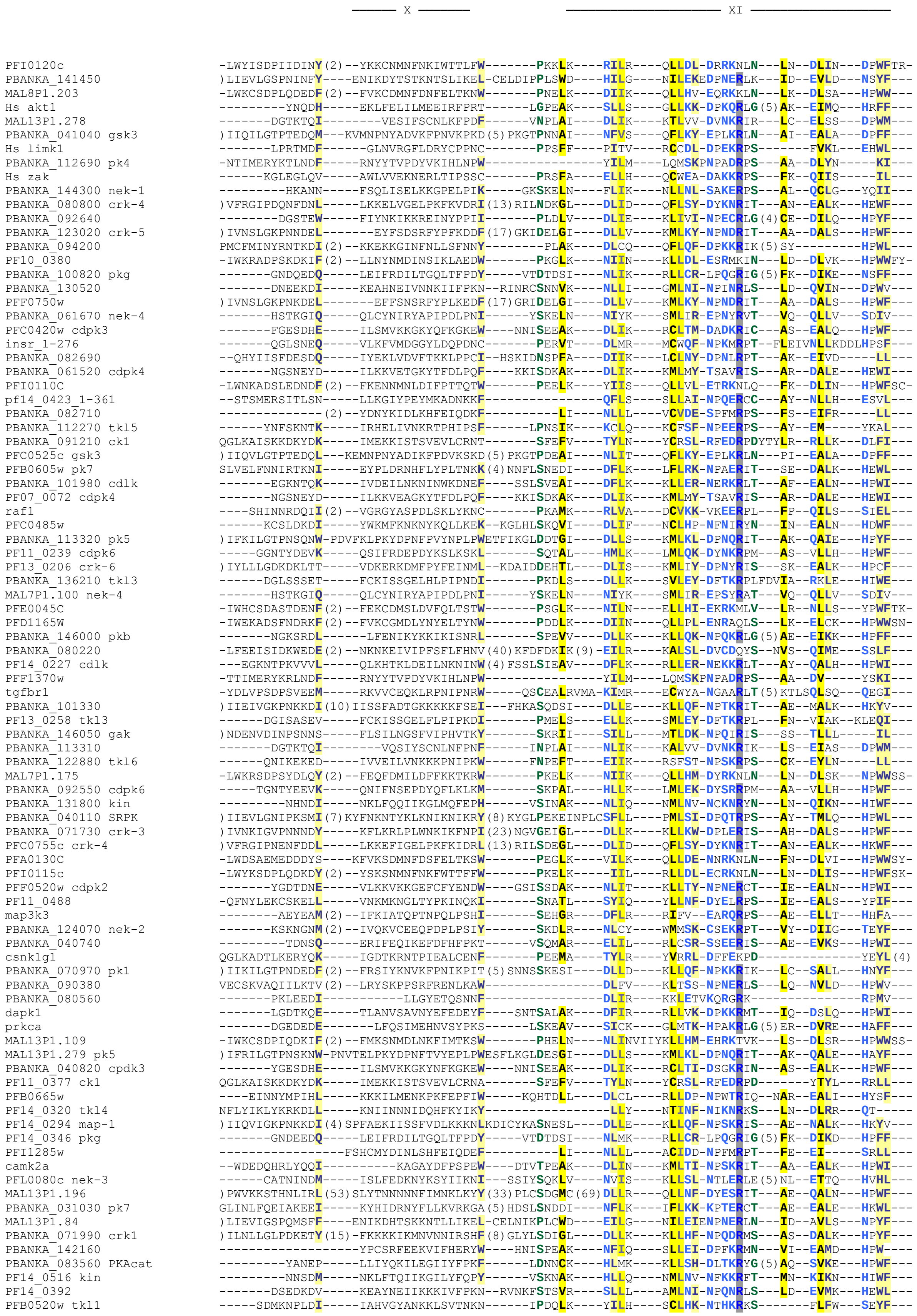

## Tewari et al., Figure S1 A (cont.)

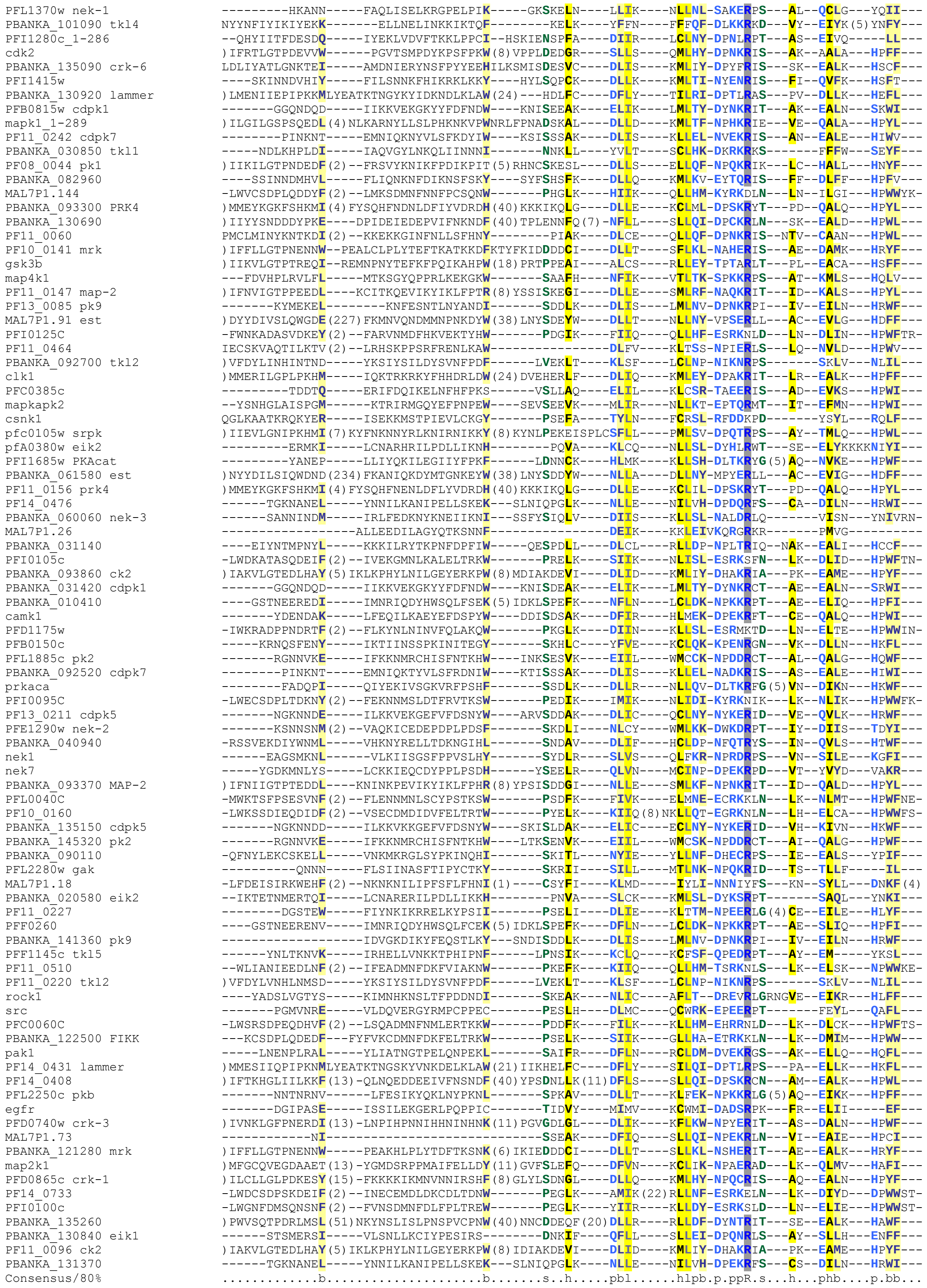

Tewari *et al.*, Fig. S1B

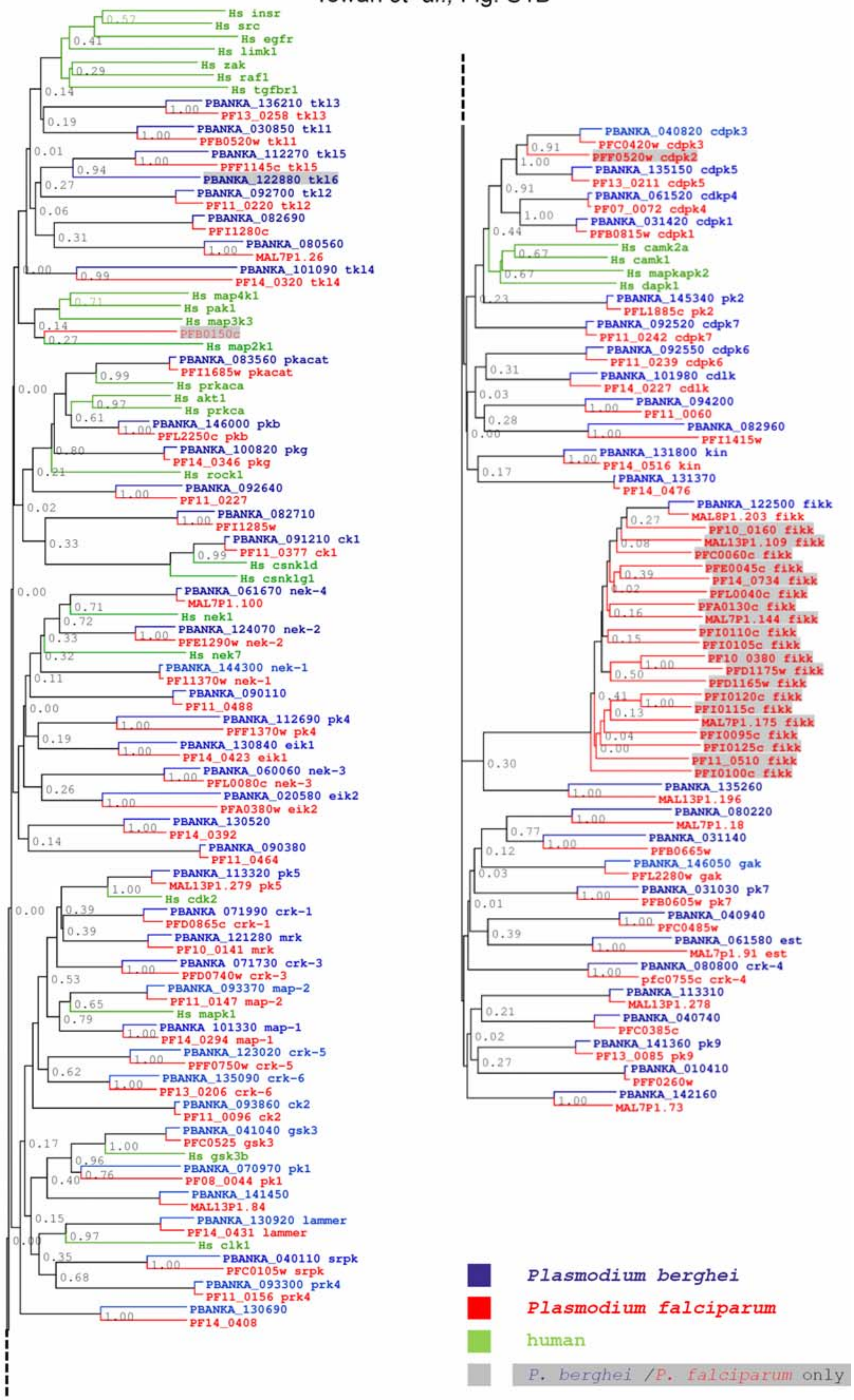

**Figure S1 (relates to Figure 1). Comparative Analysis of Kinase Domain Sequences from *P. berghei* and *P. falciparum***

(A) Multiple sequence alignment of *Plasmodium* kinase domains. The alignment was generated using the MUSCLE software (Edgar, 2004) and coloured using the CHROMA software to highlight conserved residues (Goodstadt and Ponting, 2001). Kinase domain sequences for *P. falciparum* were taken from Ward et al., (2004), those from *P. berghei* are contained in a supplemental text file entitled “Pb kinase domain sequences”.

(B) Evolutionary relationships of *Plasmodium* and selected human kinase domains. The evolutionary history was inferred from the alignment in Figure S1 using the Neighbor-Joining method (Saitou and Nei, 1987). The optimal tree with the sum of branch length = 72.77107574 is shown. The percentage of replicate trees in which the associated taxa clustered together in the bootstrap test (500 replicates) are shown next to the branches (Felsenstein, 1985). The tree is drawn to scale, with branch lengths in the same units as those of the evolutionary distances used to infer the phylogenetic tree. The evolutionary distances were computed using the Poisson correction method (Zuckerkandl and Pauling, 1965) and are in the units of the number of amino acid substitutions per site. All positions containing alignment gaps and missing data were eliminated only in pairwise sequence comparisons (Pairwise deletion option). There were a total of 4009 positions in the final dataset. Phylogenetic analyses were conducted in MEGA4 (Tamura et al., 2007).

Tewari et al., Figure S2 A (top)

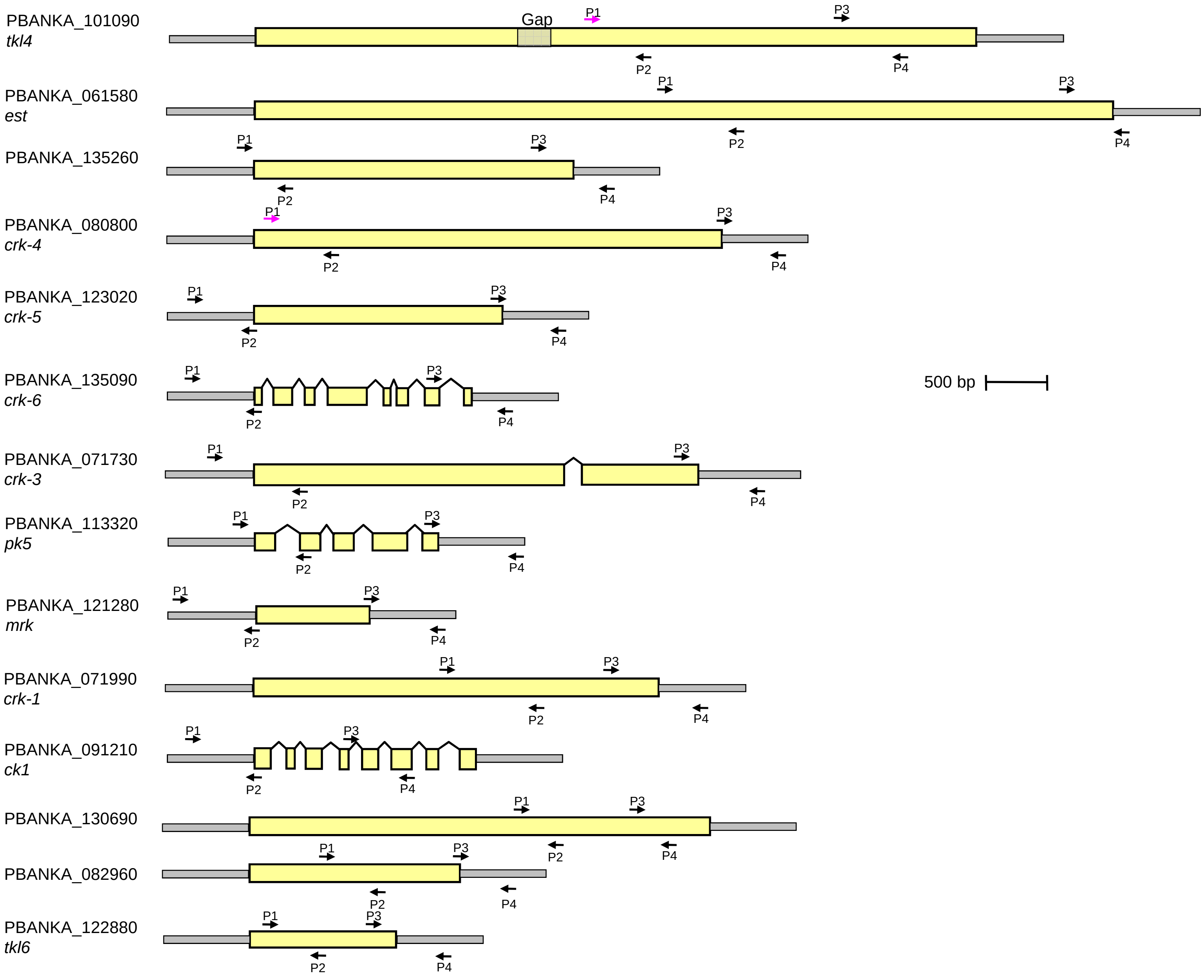

Tewari et al., Figure S2 A (cont.)

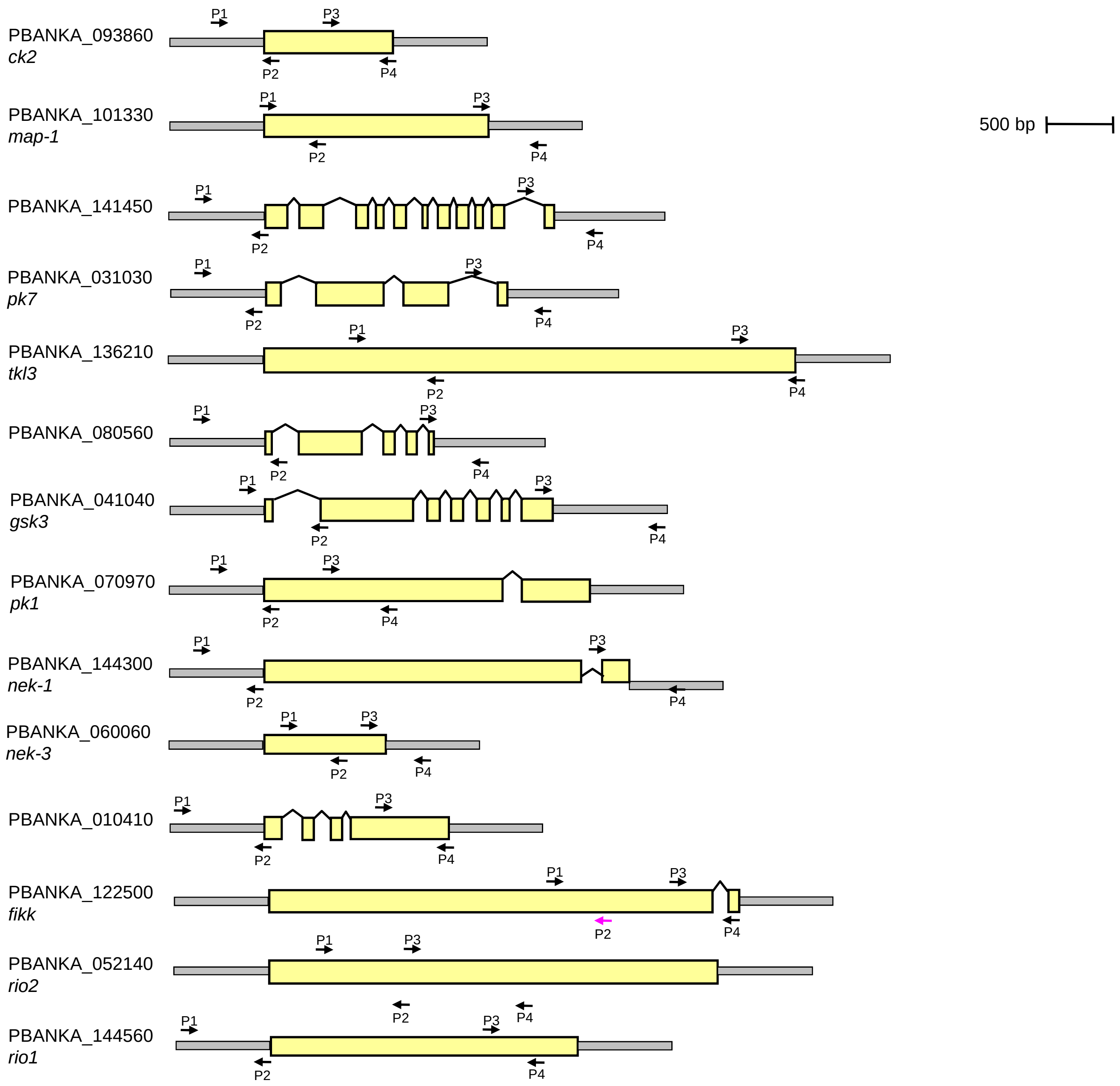

# Tewari et al., Figure S2 A (cont.)

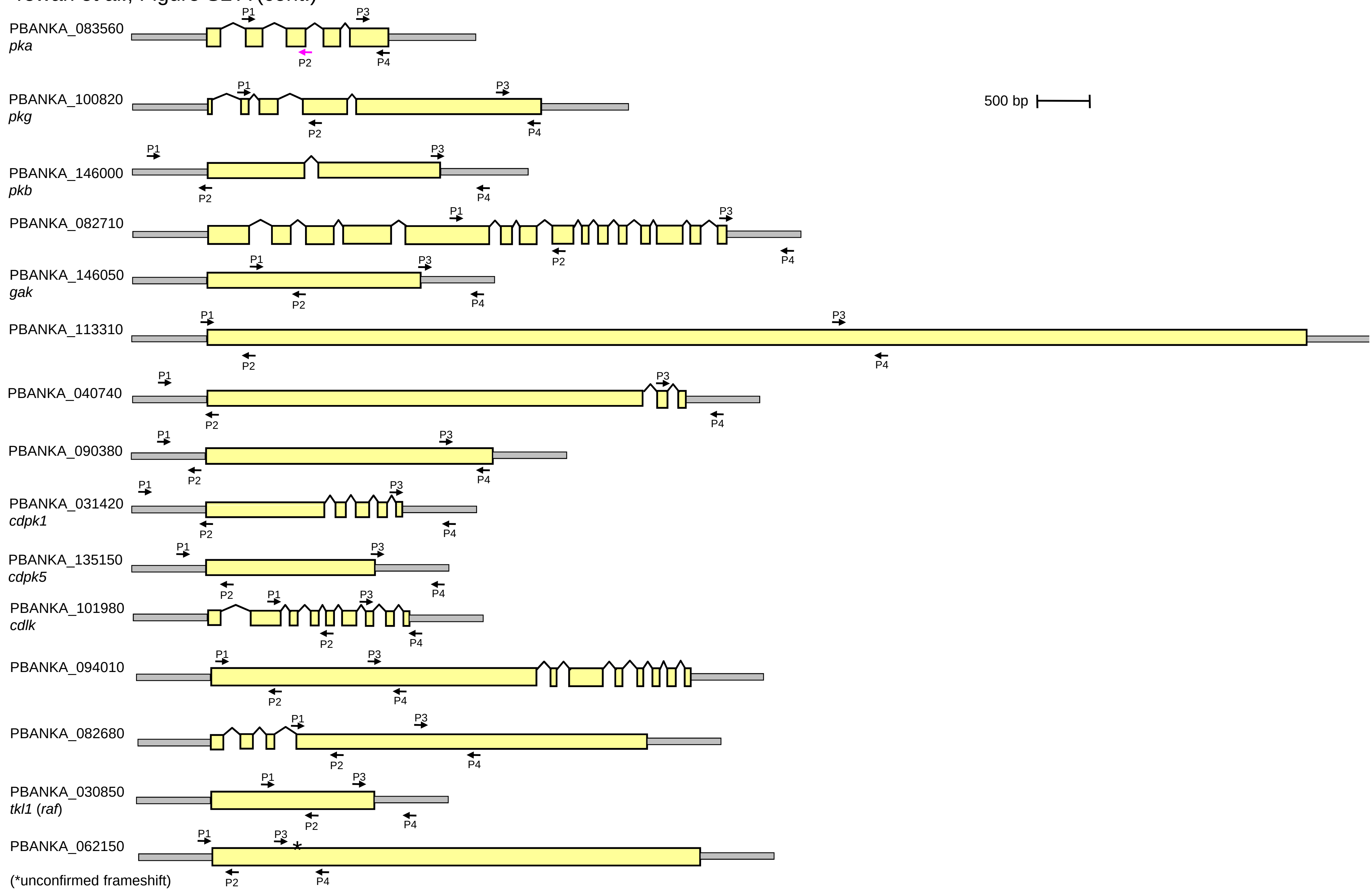

Tewari et al., Figure S2 A (cont.)

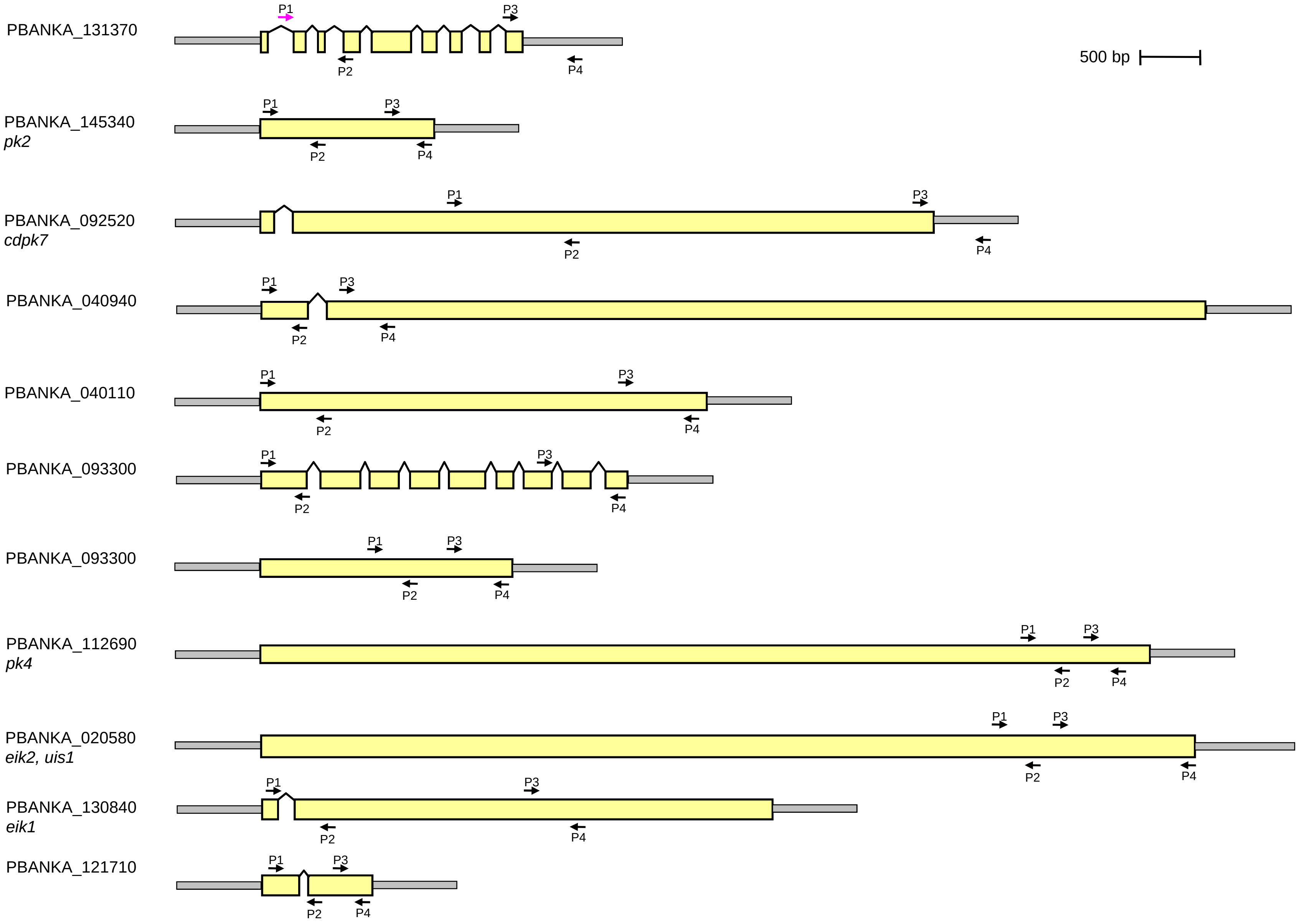

Tewari et al., Figure S2 A (cont.)

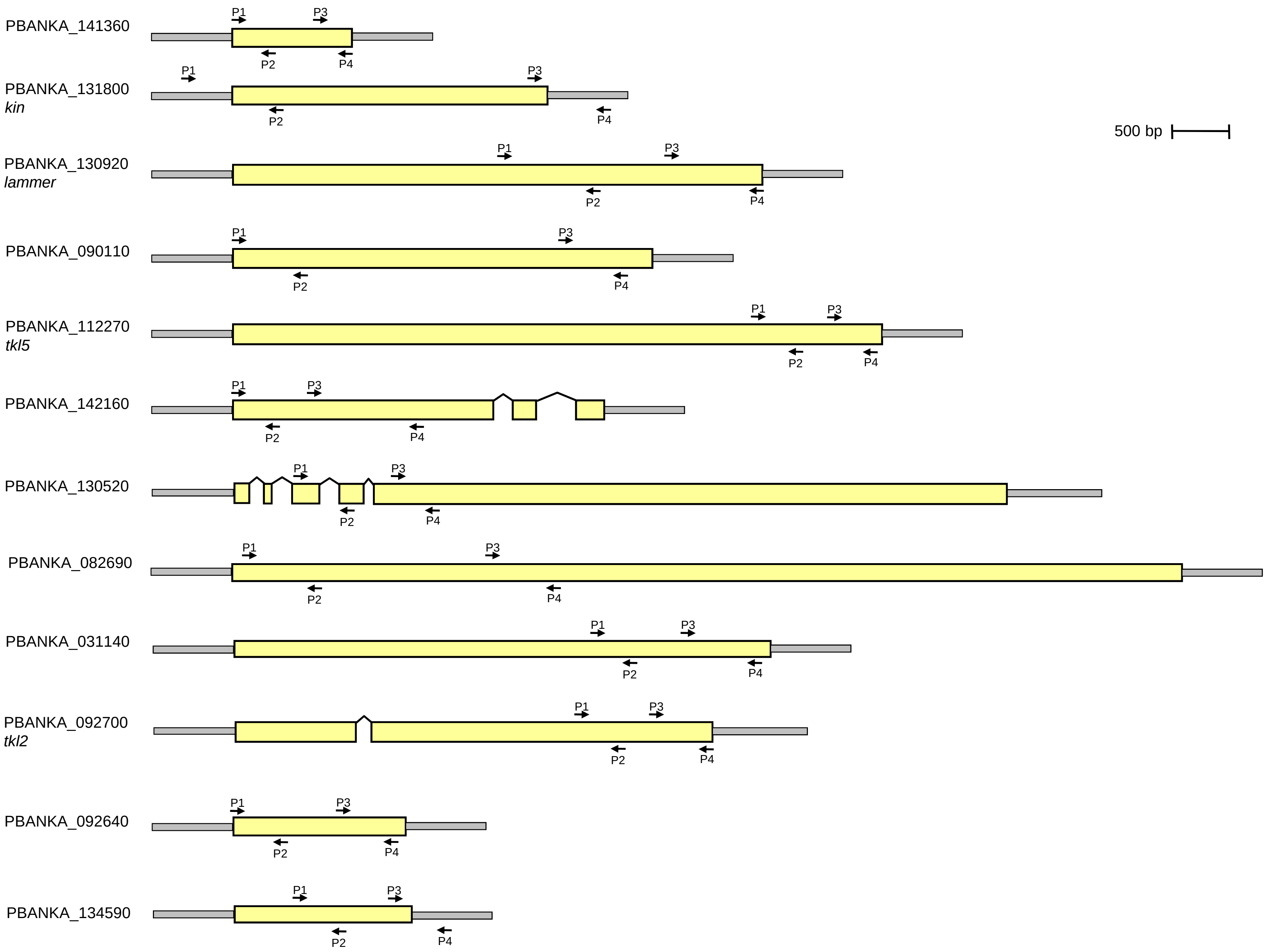

**Tewari et al., Figure S2B**

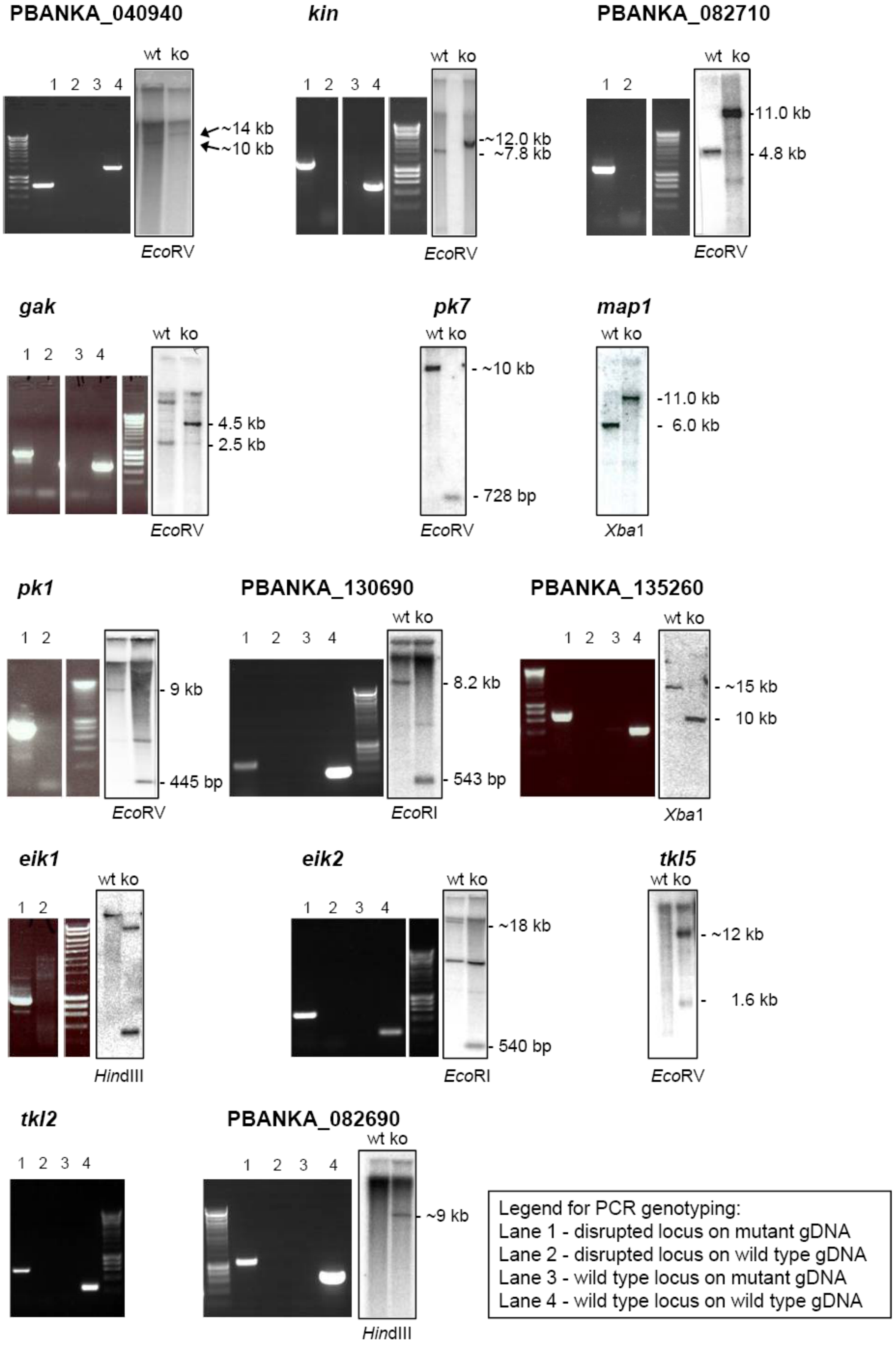

**Figure S2 (relates to Figure 2). Vector Designs and Genotyping Data**

(A) Description of gene targeting vectors. Gene models are shown as predicted in the May 2010 assembly of the *P. berghei* ANKA genome and have not been verified experimentally.

(B) PCR and Southern hybridisations confirming the disruption of 14 ePK genes. PCR genotyping (on the left) used the same rationale as illustrated in Figure 2. Southern blots (on the right) were routinely probed with gene-specific probes flanking the integration site. Diagnostic bands confirming correct integration are indicated by arrows. In a few cases evidence from only one method of genotyping was obtained. Additional details are available from the authors on request.

**Table S1 (relates to Fig.1): List of all *P. berghei* kinase-like genes and their *P. falciparum* orthologs.** Gatekeeper residues are taken from the multiple sequence alignment in Fig. S1, or from a manual alignment where indicated. Also shown are the total number of knock out attempts and how many of these led to deletion of the target as confirmed by genotype analysis. "p" gives an estimate for the likelihood of error for "possibly essential" genes. It is calculated from the average technical failure rate (0.248 per attempt for redundant genes in this study) to the power of n, where n is the total number deletion attempts for the gene. * = genes for which no mutant clones were obtained despite positive genotyping

| *Gene name* | ***P. berghei* ID** | ***P. falciparum* ortholog** | **Group** | **"Gatekeeper"** | **Knock out attempts: Total** | **Successful** | **Outcome** | **p** |
|---|---|---|---|---|---|---|---|---|
| ***cdpk1*** | **PBANKA_031420** | PFB0815w | CaMK | T | 6 | 0 | Possibly essential | 0.00024 |
| ***cdpk2*** | **no ortholog** | PFF0520w | CaMK | | | | n/a | |
| ***cdpk3*** | **PBANKA_040820** | PFC0420w | CaMK | M | 4 | 3 | KO confirmed | |
| ***cdpk4*** | **PBANKA_061520** | PF07_0072 | CaMK | S | 7 | 7 | KO confirmed | |
| ***cdpk5*** | **PBANKA_135150** | PF13_0211 | CaMK | L | 6 | 0 | Possibly essential | 0.00024 |
| ***cdpk6*** | **PBANKA_092550** | PF11_0239 | CaMK | M | 16 | 12 | KO confirmed | |
| ***cdpk7*** | **PBANKA_092520** | PF11_0242 | CaMK | M | 5 | 0 | Possibly essential* | 0.00095 |
| ***cdlk*** | **PBANKA_101980** | PF14_0227 | CaMK | L | 11 | 8 | KO confirmed | |
| ***pk2*** | **PBANKA_145340** | PFL1885c | CaMK | L | 4 | 0 | Possibly essential | 0.00382 |
| | **PBANKA_040940** | PFC0485w | CaMK | M | 5 | 3 | KO confirmed | |
| | **PBANKA_094200** | PF11_0060 | CaMK | M | | | Not done | |
| | **PBANKA_082960** | PFI1415w | CaMK | ? | 5 | 0 | Possibly essential | 0.00095 |
| | **PBANKA_031140** | PFB0665w | CaMK | M | 5 | 0 | Possibly essential | 0.00095 |
| | **PBANKA_080220** | MAL7P1.18 | CaMK | M | | | Not done | |
| ***kin*** | **PBANKA_131800** | PF14_0516 | CaMK, Snf1-like | L | 4 | 3 | KO confirmed | 0.00382 |
| | **PBANKA_131370** | PF14_0476 | CaMK, Snf1-like | L | 4 | 0 | Possibly essential | 0.00382 |
| | **PBANKA_141360** | PF13_0085 | CaMK, Snf1-like | M | 9 | 0 | Possibly essential | 3.6E-06 |
| ***pka*** | **PBANKA_083560** | PFI1685w | AGC | L | 3 | 0 | Possibly essential | 0.01535 |
| ***pkg*** | **PBANKA_100820** | PF14_0346 | AGC | T | 3 | 0 | Possibly essential | 0.01535 |
| ***pkb*** | **PBANKA_146000** | PFL2250c | AGC | L | 6 | 0 | Possibly essential | 0.00024 |
| | **PBANKA_092640** | PF11_0227 | AGC | M | 8 | 0 | Possibly essential | 1.5E-05 |
| | **PBANKA_040740** | PFC0385c | AGC | L | 3 | 0 | Possibly essential | 0.01535 |
| | **PBANKA_082710** | PFI1285w | AGC | L | 3 | 3 | KO confirmed | |
| | **PBANKA_113310** | MAL13P1.278 | orphan | L | 4 | 0 | Possibly essential | 0.00382 |
| | **PBANKA_090380** | PF11_0464 | orphan | Q | 5 | 0 | Possibly essential | 0.00095 |
| | **PBANKA_010410** | PFF0260w | orphan | M | 8 | 0 | Possibly essential | 1.5E-05 |
| ***gak*** | **PBANKA_146050** | PFL2280w | GAK | M | 4 | 3 | KO confirmed | |
| ***pk7*** | **PBANKA_031030** | PFB0605w | orphan | N | 9 | 5 | KO confirmed | |
| ***est*** | **PBANKA_061580** | MAL7P1.91 | orphan | I (ma | 4 | 0 | Possibly essential | 0.00382 |
| ***ck1*** | **PBANKA_091210** | PF11_0377 | CK1 | L | 4 | 0 | Possibly essential | 0.00382 |
| ***pk5*** | **PBANKA_113320** | MAL13P1.279 | CMGC, CRK | F | 2 | 2 | KO confirmed | |
| ***mrk*** | **PBANKA_121280** | PF10_0141 | CMGC, CRK | M | 6 | 0 | Possibly essential | 0.00024 |
| ***crk-1*** | **PBANKA_071990** | PFD0865c | CMGC, CRK | M | 3 | 0 | Possibly essential | 0.01535 |
| ***crk-3*** | **PBANKA_071730** | PFD0740w | CMGC, CRK | F | 4 | 0 | Possibly essential | 0.00382 |
| ***crk-5*** | **PBANKA_123020** | PFF0750w | CMGC, CRK | Y | 5 | 0 | Possibly essential | 0.00095 |
| ***crk-6; PK6*** | **PBANKA_135090** | MAL13P1.185 | CMGC, CRK | F | 6 | 0 | Possibly essential | 0.00024 |
| ***map-1*** | **PBANKA_101330** | PF14_0294 | CMGC, MAPK | F | 2 | 2 | KO confirmed | |
| ***map-2*** | **PBANKA_093370** | PF11_0147 | CMGC, MAPK | L | 4 | 3 | KO confirmed | |
| ***ck2*** | **PBANKA_093860** | PF11_0096 | GMGC, CK2 | F | 4 | 0 | Possibly essential | 0.00382 |
| ***gsk-3*** | **PBANKA_041040** | PFC0525c | CMGC, GSK3 | M | 9 | 0 | Possibly essential | 3.6E-06 |
| ***pk1*** | **PBANKA_070970** | PF08_0044 | CMGC, GSK3 | M (ma | 4 | 3 | KO confirmed | |
| | **PBANKA_141450** | MAL13P1.84 | CMGC, GSK3 | F | 5 | 0 | Possibly essential | 0.00095 |
| ***lammer*** | **PBANKA_130920** | PF14_0431 | CMGC, SRPK | F | 3 | 0 | Possibly essential | 0.01535 |
| ***srpk*** | **PBANKA_040110** | PFC0105w | CMGC, SRPK | F | 5 | 4 | KO confirmed | |
| ***prk4*** | **PBANKA_093300** | PF11_0156 | CMGC, SRPK | F | 4 | 0 | Possibly essential | 0.00382 |
| | **PBANKA_130690** | PF14_0408 | CMGC, SRPK | T | 3 | 3 | KO confirmed | |
| ***crk-4*** | **PBANKA_080800** | PFC0755c | CMGC | ? (po | 4 | 0 | Possibly essential | 0.00382 |
| | **no ortholog** | PFB0150c | CMGC | | | | n/a | |
| | **PBANKA_135260** | MAL13P1.196 | CMGC | F | 2 | 2 | KO confirmed | |
| ***nek-2*** | **PBANKA_124070** | PFE1290w | CMGC, NEK | M | 7 | 6 | KO confirmed | |

| | | | | | | | | |
|---|---|---|---|---|---|---|---|---|
| ***nek-4*** | **PBANKA_061670** | MAL7P1.100 | CMGC, NEK | M | 2 | 2 | KO confirmed | |
| ***nek-1*** | **PBANKA_144300** | PFL1370w | CMGC, NEK | M | 8 | 0 | Possibly essential | 1.5E-05 |
| ***nek-3*** | **PBANKA_060060** | PFL0080c | CMGC, NEK | I | 7 | 0 | Possibly essential | 5.9E-05 |
| ***eik1*** | **PBANKA_130840** | PF14_0423 | CMGC, ELK | M | 4 | 2 | KO confirmed | |
| ***eik2; uis1*** | **PBANKA_020580** | PFA0380w | CMGC, ELK | M | 5 | 3 | KO confirmed | |
| ***pk4*** | **PBANKA_112690** | PFF1370w | CMGC, ELK | M | 3 | 0 | Possibly essential | 0.01535 |
| | **PBANKA_090110** | PF11_0488 | orphan | M | 9 | 0 | Possibly essential | 3.6E-06 |
| | **PBANKA_130520** | PF14_0392 | orphan | M | 6 | 0 | Possibly essential* | 0.00024 |
| | **PBANKA_142160** | MAL7P1.73 | orphan | L | 3 | 0 | Possibly essential | 0.01535 |
| ***tkl6*** | **PBANKA_122880** | no ortholog | TKL | L | 5 | 0 | Possibly essential | 0.00095 |
| ***tkl5*** | **PBANKA_112270** | PFF1145c | TKL | L | 3 | 3 | KO confirmed | |
| ***tkl2*** | **PBANKA_092700** | PF11_0220 | TKL | Y | 4 | 1 | KO confirmed | |
| ***tkl1 (raf)*** | **PBANKA_030850** | PFB0520w | TKL | L | 3 | 0 | Possibly essential | 0.01535 |
| ***tkl3*** | **PBANKA_136210** | PF13_0258 | TKL | L | 5 | 0 | Possibly essential* | 0.00095 |
| ***tkl4*** | **PBANKA_101090** | PF14_0320 | TKL | M | 3 | 0 | Possibly essential | 0.01535 |
| | **PBANKA_082690** | PFI1280c | orphan | T | 3 | 2 | KO confirmed | |
| | **PBANKA_080560** | MAL7P1.26 | orphan | F | 5 | 0 | Possibly essential | 0.00095 |
| | **PBANKA_121710** | PFC0945w | pseudokinase | n.d. | 5 | 0 | Possibly essential | 0.00095 |
| | **PBANKA_094010** | PF11_0079 | pseudokinase | n.d. | 4 | 0 | Possibly essential | 0.00382 |
| | **PBANKA_082680** | PFI1275w | pseudokinase | n.d. | 4 | 0 | Possibly essential | 0.00382 |
| | **PBANKA_134590** | PF13_0166 | pseudokinase | n.d. | 3 | 0 | Possibly essential | 0.01535 |
| | **PBANKA_062150** | MAL7P1.127 | pseudokinase | n.d. | 5 | 0 | Possibly essential | 0.00095 |
| ***rio1*** | **PBANKA_144560** | PFL1490w | aPK, RIO | | 3 | 0 | Possibly essential | 0.01535 |
| ***rio2*** | **PBANKA_052140** | PFD0975w | aPK, RIO | | 4 | 0 | Possibly essential | 0.00382 |
| ***fikk*** | **PBANKA_122500** | MAL8P1.203 | FIKK, ancestral | S | 5 | 0 | Possibly essential | 0.00095 |
| | **no ortholog** | MAL7P1.144 | FIKK | | | | n/a | |
| ***R45*** | **no ortholog** | PFD1175w | FIKK | | | | n/a | |
| | **no ortholog** | PFI0095c | FIKK | | | | n/a | |
| | **no ortholog** | PFI0100c | FIKK | | | | n/a | |
| | **no ortholog** | PFI0105c | FIKK | | | | n/a | |
| | **no ortholog** | PFI0110c | FIKK | | | | n/a | |
| | **no ortholog** | PFI0115c | FIKK | | | | n/a | |
| | **no ortholog** | PFI0120c | FIKK | | | | n/a | |
| | **no ortholog** | PFI0125c | FIKK | | | | n/a | |
| | **no ortholog** | PFL0040c | FIKK | | | | n/a | |
| | **no ortholog** | PFD1165w | FIKK | | | | n/a | |
| | **no ortholog** | PFE0045c | FIKK | | | | n/a | |
| | **no ortholog** | PFC0060c | FIKK | | | | n/a | |
| | **no ortholog** | PF14_0734 | FIKK | | | | n/a | |
| | **no ortholog** | PFA0130c | FIKK | | | | n/a | |
| | **no ortholog** | PF11_0510 | FIKK | | | | n/a | |
| | **no ortholog** | PF10_0160 | FIKK | | | | n/a | |
| | **no ortholog** | MAL7P1.175 | FIKK | | | | n/a | |
| | **no ortholog** | PF10_0380 | FIKK | | | | n/a | |
| | **no ortholog** | MAL13P1.109 | FIKK | | | | n/a | |
| | | | | | | | **sum:** | **0.23139** |

## Table S2. Unique ePKs of *P. berghei*/*P. falciparum* and Their Distribution across Other *Plasmodium* Genomes

| | ***P. berghei*** | ***P. yoelii*** | ***P. chabaudi*** | ***P. vivax*** | ***P. knowlesi*** | ***P. falciparum* 3D7** | ***P. reichenowi*** | ***P. gallinaceum*** |
|---|---|---|---|---|---|---|---|---|
| ***tkl6*** | PBANKA_122880 | PY04849 PY04848 | PCAS_122950 | PVX_093670 | PKH_011700 | — | — | Pgal0325e01.p1k Pgal0616c03.p1k Pgal0616c03.q1k Pgal0489d11.p1k |
| **PFB0150c** | — | — | — | PVX_003590 | PKH_041680 | PFB0150c | 3502696. c000217971. Contig1 | gal28a.d0000062 93. Contig1 |
| ***cdpk2*** | — | — | — | — | — | PFF0520W | 3502696.c000218538. Contig1 | gal28a.d0000019 39. Contig1 |
| **Ancestral *fikk*** | PBANKA_122500 | PY03326 | PCAS_122560 | PVX_088265 | PKH_011260 | MAL8P1.203 | 3502696.c000024654. Contig1 | gal28a.d0000159 42. Contig1 |
| ***fikk* expansion** | none | none | none | none | none | 20 | yes | none |

**Table S3 (relates to Fig. 3). Data from phenotyping 23 mutants.** All data are given as percentage of wild type controls studied in parallel. SD = standard deviation. n = number of replicate experiments.

| Gene name | *P. berghei* ID | Ookinete conversion % of control | *SD* | n | Oocyst numbers % of control | *SD* | n | Oocyst prevalence % of control | *SD* | n | Oocyst sporozoites % of control | *SD* | n | Salivery gland sporozoites % of control | *SD* | n | Transmission by mosquito bite |
|---|---|---|---|---|---|---|---|---|---|---|---|---|---|---|---|---|---|
| ***cdpk3*** | PBANKA_040820 | **94.65** | *6.97* | 3 | **3.30** | *2.79* | 8 | n.d. | | | n.d. | | | **0.00** | *0.00* | 2 | n.d. |
| ***cdpk4*** | PBANKA_061520 | **0.00** | *0.00* | 4 | **0.00** | *0.00* | 4 | 0.00 | *0.00* | 4 | n.d. | | | **0.00** | *0.00* | 4 | n.d. |
| ***cdpk6*** | PBANKA_092550 | **90.41** | *11.70* | 3 | **91.20** | *11.94* | 3 | **98.35** | *5.79* | 3 | **152.22** | *19.18* | 3 | **28.35** | *44.69* | 3 | transmitted (delayed) |
| ***cdlk*** | PBANKA_101980 | **84.90** | *7.63* | 6 | **104.66** | *17.48* | 3 | **102.79** | *13.03* | 3 | **41.66** | *18.50* | 3 | **4.16** | *1.70* | 3 | transmitted |
| | PBANKA_040940 | **70.59** | *14.26* | 3 | **49.04** | *23.30* | 6 | **97.36** | *16.94* | 6 | **132.61** | *18.13* | 3 | **9.98** | *2.11* | 6 | no transmission (0/3) |
| ***kin*** | PBANKA_131800 | **89.00** | *6.76* | 3 | **105.28** | *29.73* | 3 | **103.85** | *0.00* | 3 | **148.56** | *27.35* | 3 | **4.65** | *1.03* | 3 | delayed to 15d |
| | PBANKA_082710 | ***97.11*** | *7.82* | 2 | **91.58** | *2.23* | 2 | **95.56** | *6.29* | 2 | **52.93** | *0.27* | 2 | **51.24** | *16.37* | 2 | transmitted |
| ***gak*** | PBANKA_146050 | **4.62** | *1.54* | 3 | **3.28** | *3.60* | 6 | **47.98** | *32.20* | 6 | **0.00** | *0.00* | 3 | **0.00** | *0.00* | 3 | n.d. |
| ***pk7*** | PBANKA_031030 | **8.92** | *3.70* | 8 | **8.88** | *5.59* | 4 | **63.45** | *8.63* | 4 | **0.00** | *0.00* | 7 | **0.00** | *0.00* | 3 | n.d. |
| ***pk5*** | PBANKA_113320 | **87.37** | *14.24* | 3 | **56.43** | *2.28* | 3 | **94.44** | *9.62* | 3 | **107.63** | *18.95* | 3 | **65.27** | *16.06* | 3 | transmitted |
| ***map-1*** | PBANKA_101330 | **93.68** | *15.82* | 3 | **63.33** | *23.43* | 3 | **87.72** | *12.15* | 3 | **92.97** | *18.31* | 3 | **101.53** | *23.25* | 3 | transmitted |
| ***map-2*** | PBANKA_093370 | **0.00** | *0.00* | 3 | **0.00** | *0.00* | 3 | **0.00** | *0.00* | 3 | n.d. | | | **0.00** | *0.00* | 3 | n.d. |
| ***pk1*** | PBANKA_070970 | **98.40** | *8.84* | 3 | **87.36** | *31.51* | 3 | **104.00** | *6.97* | 3 | **98.82** | *13.24* | 3 | **104.79** | *25.44* | 3 | transmitted |
| ***srpk*** | PBANKA_040110 | **0.00** | ***0.00*** | 3 | **0.00** | *0.00* | 3 | **0.00** | *0.00* | 3 | n.d. | | | n.d. | | | n.d. |
| | PBANKA_130690 | **98.50** | *9.17* | 3 | **106.15** | *15.51* | 3 | **100.00** | *6.66* | 3 | **128.00** | *45.67* | 3 | **124.94** | *16.37* | 3 | transmitted |
| | PBANKA_135260 | **95.79** | *10.15* | 3 | **57.46** | *9.47* | 3 | **103.70** | *8.49* | 3 | **136.00** | *28.09* | 3 | **66.41** | *6.58* | 3 | transmitted |
| ***nek-2*** | PBANKA_124070 | **0.00** | *0.00* | 3 | **0.00** | *0.00* | 3 | **0.00** | *0.00* | 3 | n.d. | | | n.d. | | | n.d. |
| ***nek-4*** | PBANKA_061670 | **0.00** | *0.00* | 3 | **0.00** | *0.00* | 3 | **0.00** | *0.00* | 3 | n.d. | | | n.d. | | | n.d. |
| ***eik1*** | PBANKA_130840 | **84.47** | *11.65* | 3 | **109.68** | *55.03* | 3 | **102.44** | *26.38* | 3 | **108.54** | *11.61* | 3 | **94.40** | *26.16* | 3 | transmitted |
| ***eik2; uis1*** | PBANKA_020580 | **51.87** | *19.40* | 3 | **19.86** | *15.46* | 3 | **64.22** | *44.57* | 3 | n.d. | | | **14.89** | *10.80* | 3 | transmitted (4/4) |
| ***tkl5*** | PBANKA_112270 | **83.08** | *11.10* | 3 | **77.46** | *36.88* | 3 | **100.00** | *5.97* | 3 | **144.73** | *26.40* | 3 | **102.34** | *19.33* | 3 | transmitted |
| ***tkl2*** | PBANKA_092700 | **79.45** | *13.07* | 3 | ***124.24*** | *15.96* | 3 | **121.46** | *2.53* | 3 | **92.46** | *5.29* | 3 | **83.12** | *10.89* | 3 | transmitted |
| | PBANKA_082690 | **83.11** | *3.45* | 3 | **84.21** | *9.25* | 3 | **94.83** | *7.90* | 3 | **84.55** | *11.57* | 3 | **81.98** | *8.77* | 3 | transmitted |

**Table S4. *P. berghei* Protein Kinase Domain Sequences Used in This Study**

>PBANKA_101090 PbTKL4
ITFLKKINEHGNGKIFLCSYNIFNNKFFIIKLIDIANNINENYIFKNIFNEVKCLLTFQNSKKGICQMYSYGIIP
NCNNNGFTYYLLMKYYEGNLKDLMNYAHISYLKEIKKIRNNAKQFFLPIVCKKRTEEIILNFKIAKITKKITKRC
KGNFIFCYKYLCRYTNVYSCYTMILKNRIYIKIIQLKFILFILNIFIQIIEQIIYVHKKKIIHFDINTCNILINF
KKTYPLLMSKKYKIKKNNYINNCNTKQTLKNIQSQIVMRDDFVENERKIYLNFIPPSYQINNSIYKKQNKFYNNF
DTSMASFHYRQYFKIIMKKKDCQHIAIPSIVISDFGESKFFLRDTDFIFFRKNRGNEILAAPELLINKNKYIYSK
NKYKIEKKNGKEDTEIKSRKAIQDSFYFENIPQNIIFQNEQSEMNYFYSTITCFENNCKFSCLNTKNDKIRILRY
NNINMKKSLRKNSIVSLEFYKYLKKIKMLIKKKLGNQKKLQKKKINMSKSDIWSLGSLLYEMITNESLFNYYNFI
YIKIYEKKELLNELINKKIKTQFKELKYFFNFFFQFDLKKRKDVYEIYKESIKIYNFY
>PBANKA_061580 PbEST
LKLMHIIFGKKYKCYKNENDKMCIGLCYEKLKKIKIKLNNSVIEIKYPKFDYIYDNIKAYNCGLTESECKFLFFQ
IVNGISFLQTCYQSNIIRLTDIKLQNILVFTDIYNVYNPIKWHLCISDFGCSAMEYATFYLENSKNYINAYKTLL
NQWKYQFKNQLSSYFQGTVYTMAPEGLCYDHMGNFRQSKYNKLIYFYEQNFGNIEDRFQELQMPLVSIKNSINNF
NILNVNSSSNFHFLEKKDGKYEMNNTYNDMDADKNGSEVCKGETNKNSKKKSTNKNVKANESANSNGNNTSSKKK
GKVEEENNDKKETSDKINLTENNKMNSSPEYLPFDVRSDSWSLGIILADLGKCGIGSYEYMISEKGNGNNNISKG
NIIDLNNIILNDLNILDDEENFYLQYIMNYYDILSIQWDNDEIENDQVNKNADRNKKRDNSEMENKRNNDNSNNN
GCTNNNYSKKQKNKSLENNNSKEDVPGNNCFVIDNANESISNEVSFCKDKPNAHNNNYDIKEETNNNKNAVLNNQ
IKSTLNFDECNKIKGNNDNSMKSLELFTKYYKSFILKYKDYIKIDKIGYIKNEFVKYYKLYKHMMNENNIERILL
LLFLIIINNLTYNYSCETQSFLKIELTIKNIGSGIFKFRNKKEKERYLQEFKANIQKDYMTGNKEYWNSRLTNKY
LAVILKDVCYDNFANRKKGIKKCFNKFEYPLNYSDDYWNLLADLLNYMPYERLLACEVIGHDFF
>PBANKA_135260
YSIQNKLGAGAYGEIWYGINLNKNVPFQNVVLKKIFIKKSVDESEQNLNDDEREKEYEIYAMREVYFGEIFKNCD
NISRYIEHFKESETSENNKEHITFIWIVFANEGYSLSQHLFETDKNNSGMIIPKLWWSIKKQNIGMLVIKDLMHQ
LLNGINIAHKKHITHRDIKMENIFVSPNTPFTVRIGDWGSAVEYKNKSFFFTPSMEEETEGYQPPESLFGHMKKN
FMRLPYYDMWGIGILFLQFVLGTKNPLEIKNKRNEMKLKRIYSKYSKDILKEVIFIQGLSDLCLIPWVSQTPDRL
MSLYDMKNNEKNSLQYSSIYGRNLIINKLEYFAITKNLIEIKRKRYNLINNMMSNKYNSLISLPNSPVCPNWKCL
HKYDEQANYNNKYNNNTFDKIQNKFLQQKKENYTFFKNNCDDEQFQKILQERDPSGVGLPDKNARDLLRRLLDFD
YNTRITSEEALKHAWF
>PBANKA_080800 PbCRK-4
YKIVKKLGEGVYGKVFKAESLEDSYLNFAVKVLRYFWPNFKYKFGSEEFAINEFNIMRILFHPNVVCLLDSFRVH
TYRKNKIKNHRGQKTRCETLSAEYDFSFQRHRRMEKVQYSPSRETIERNNKYRNIVSKSCLTIEELEKNLVLNSF
DKEATIQDKGNLYNNHTGKVDETSTIFSNNNCVKVLNYDEENNNLDNNKSNVLSSKMQRDIKRERSGDIFQYYPL
KKKMKTSNYKNGKRNEAVKVMNKSIDKLKFRKHSKKLKKIENKNDDYIENWDLFLVIEKCDCSLNDILNKAKKKH
FSFIQDIKKDTTKCLPSERIDLSYDHLHNYVKYVYLPLKKIENRYFYPDMPSLTEMQTKVIIYQMLQGINHFHKK
FIIHRDIKPANTLIKNIQYLSDGLNDSKEWIVKIADFGLGVYDHFLKTETKDCNIITLQYRPPEILCNSTLYNYS
VDIWSIGITMCECLLGFVPVTSKFESSVLFKILVFRGIPDQNFDNLLKKELVGELPKFKVDRIKMLEIIFTDIYG
RRILNDKGLDLIDQFLSYDYKNRITANEALKHEWF
>PBANKA_123020 PbCRK-5
VSLEEKLGGGTYGDVYKGKVVKYNNNNSDLYQNTNYLPYDYYTDEFIFFRKNIYAIKFFKDDLRTINEEGISCTT
LRELSCLKNIGRHPNILRLIDVTIDRQKGISEYINRQILQHYTSNYHHQVKIDMVPLSLDQKFIFAAYEYCDGGD
LKKLIQKTKISENQAGLCLKEAKWLSFQLLNGLAYLHNNKMCHRDLKPENVMLQYTHNNKYLLKIGDLGLCRELK
NDGDMTPTVCTIYYRPLEVLLSKFDRSKTRSGKNGVNKSGSGSGSAKNGSVKNGSVKNGSVKNGSVKNGSVKNGS
VKNGSVKNGSVKNGSIKNGSIKNGSIKNGSIKNGSGRCSEGRRSDDAYMGDEAFEKDYESFNDRDFQYGLNVDIW
SAACIICELIIGKPLFRGVTEFDLIIRIVNSLGKPNNDELEYFSDSRFYPFKDDFFNIKIKNRKDALNVITNGKI
DELGIDLLVKMLKYNPNDRITAADALSHPWF
>PBANKA_135090 PbCRK-6
FDFMDIIGKGTYGVVYKAIDKKENKIVAIKKIINLCDHNYGISKIILRELSILQKINHKNIIVLRNIFYGKDIEQ
KLIGENLENSCLYLTFEYCDIDLLNFTKIYNLNIKEVKYIIFEILLALCYLHSNNYLHRDIKPENIFINSKGEIK
LGDLGLSVEKSDNMTPSVVTIWYRSPELLLKQNNYDQKIDIWSLGCLFVELITGRPLFPGKNDQSQLDLIYATLG
NKTEIAMDNIERYNSFPYYEEHILKSMISDESVCDLISKMLIYDPYFRISSKEALKHSCF
>PBANKA_071730 PbCRK-3
IVKIHQVGQGAYGDVWMAEDIENNKRIALKKLKLNGNKEGLAKTYIREISILNSLQHKNVVELIGVIRTNIIPEE
IKSNNILYNFEKKNQLLNYQNVKDKSIDSYIMPDRALKKKLAKCSSIYQSSESSESSESSSDSSDGISCDSDSCL
CNKNPLNKKVNSCASIWMVFEYVPFDLSGYSELLREERQQKDRYKNMNLFTIGEIKNIMLQLFQALEYCHKNNVI
HRDIKIANLLIDANGILKLADFGLARFHIDSFASNMTNRVITLWYRPPELLLGSENYSSSVDMWSCGCVLGELLT
SNPLFTADNESDILKTIVNKIGVPNNNDYKFLKRLPLWNKIKFNPIHPSNINNLNQTKKIETEYIIKNLNGVGEI
GLDLLKKLLKWDPLERISAHDALSHPWF
>PBANKA_113320 PbPK5

YHGLEKIGEGTYGVVYKAQNSDGESFALKKIRLEKEDEGIPSTAIREISILKELRHSNIVKLYDVIHAKKRLILV
FEHLDQDLKKLIDVCDGGLESVTAKSFLLQLLNGIAYCHEHRVLHRDLKPQNLLINREGELKIADFGLARAFGIP
ARRYTHEVVTLWYRAPDILMGSKKYSTPIDIWSVGCIFAEMVNGRPLFPGASETDQLMRIFKILGTPNSQNWPDV
FKLPKYDPNFPVYNPLPWETFIKGLDDTGIDLLSKMLKLDPNQRITAKQAIEHPYF
>PBANKA_121280 PbMRK
IFKPNFLGEGSYGKVYKAYDTVLKKEVAIKKMKLNKISNYIDECGINFLILREIKIMKEIKHKNVMNALDLYCEK
DYINLVMEIMDYDLAKLINRKILLTDSQKKCILLQILNGLNTLHKYYFMHRDLSPANIFINKKGEVKIADFGLSS
KYAFDMHSGKMANDKYSKRALNLTSKVVTLWYRAPELLMGSNKYNSSIDMWSFGCIFAELLLQKALFPGENEIDQ
LGKIFFLLGTPNENNWPEAKHLPLYTDFTKSNKKNLKNIIKIEDDDCIDLLTSLLKLNSHERITAEEALKHRYF
>PBANKA_071990 PbCRK-1
YKKLNKISEGTYGTVFRAQNKKTKKIIALKQLKNFSNIRHEGFAITSLREINILLQLDHENILSIKEVIVGKHLN
DIYLVMEYIEHELKMLLDNKSPGFTVSELKCLLKQLLNGVNYLHTNWVMHRDLKPTNLLYSNKGILKICDFGMAR
KFSHVDNPNFTKNVVTLWYRAPELLLGEKCYTNKIDMWSIGCIFAEMILKKPLFLGENEVDQMWKILNLLGLPDK
ETYPKFYEYSFISKNKDLFKKKKIKMNVNNIRSHFPNIASQFSGLYLSDIGLDLLKKLLHFNPQDRMSASDALKH
PYF
>PBANKA_091210 PbCK1
YALGKKLGSGSFGDIYVAKDIVTMEEFAVKLESTRSKHPQLLYESKLYKILGGGIGVPKVYWYGIEGDFTIMVLD
LLGPSLEDLFTLCNRKFSLKTVLMTADQMLNRIEYVHSKNFIHRDIKPDNFLIGRGKKVTLIHIIDFGLAKKYRD
SRSHTHIPYKEGKNLTGTARYASINTHLGIEQSRRDDIEALGYVLMYFLRGSLPWQGLKAISKKDKYDKIMEKKI
STSVEVLCRNTSFEFVTYLNYCRSLRFEDRPDYTYLRRLLKDLFI
>PBANKA_093860 PbCK2
YEILKKVGRGKYSEVFNGYDTEYNRLCAIKVLKPVKKKKIKREIKILQNLHGGPNIIKLLDIVKDPVTKTPSLIF
EYINNIDFKTLYPKFTDKDIRYYIYQILKALDYCHSQGIMHRDVKPHNIMIDHENKQIRLIDWGLAEFYHPGQEY
NVRVASRYYKGPELLIDLQLYDYSLDIWSLGCMLAGMIFKKEPFFCGHDNYDQLVKIAKVLGTEDLHAYLKKYNI
KLKPHYLNILGEYERKPWSHFLNQSNMDIAKDEVIDLIDKMLIYDHAKRIAPKEAMEHPYF
>PBANKA_101330 PbMAP-1
YDIIKKIGKGAYGIVFKARCRKYKKIVAVKKIFGAFQNSTDAQRTFREIMFLHQLNGHDNIIKLLDVMRAKNDQD
IYLVFEYMETDLHEVIRADILEEVHKKYIIYQLLRALKYMHSGLLLHRDIKPSNILLNSECHLKICDFGLARSIS
TEVNENKIPVLTDYVATRWYRAPDILLGSTNYTEGVDMWSLGCIMAELLLGKPLFRGNSTMNQLEKIIEIVGKPN
KKDIEDIKSPYAETIISSFADTGKKKKKFSEIFHKASQDSIDLLEKLLQFNPTKRITAEMALKHKYV
>PBANKA_093370 PbMAP-2
YIIKHLIGRGSYGYVYLAYDKNTEKNVAIKKVNRMFEDLIDCKRILREITILNRLKSDYIIRLYDLIIPDDLLKF
DELYIVLEIADSDLKKLFKTPIFLTEEHIKTILYNLLLGENFIHESGIIHRDLKPANCLLNQDCSVKVCDFGLAR
TINSEKDTNIVNDLEENEEPGPHNKNLKKQLTSHVVTRWYRAPELILLQENYTKSIDIWSTGCIFAELLNMLQSH
INDPTNRFPLFPGSSCFPLSPDRNSKKVHEKSNRDQLNIIFNIIGTPTEDDLKNINKPEVIKYIKLFPHRKPINL
KQKYPSISDDGINLLESMLKFNPNKRITIDQALDHPYL
>PBANKA_141450
YKLIKLIGKGTFGKVYYAIDLSTQEPVAIKRSPKWRNKVSREVDLLKKMNYSKNIVNTKSIFYTTTDKGFRIQNI
VFKYMTYSLGKFIRLKKQEKRENNQICLGIKNLHEHNVAHRDLKPDNILINLDTPDINVEICDLGSAKKVEKSII
SIPYICSRWYRAPELLCGSMYYTTDVDLWSLGCIIFELINLCPLFPGKFKKDEYSEECSQIVNLIEVLGSPNINF
YENIKDYTSTKNTSLIKELCELDIPPLSWDHILGNILEKEDPNERLKIDEVLDNSYF
>PBANKA_031030 PbPK7
YKIVKTIHEGKYSKIIMCDKDGELYALKKYEKVFLEKKREFQKKTKDNKVIIKTKYDDLKNELKIITDIKNEYCL
SCKEIITNNDEVYIVNKYMENESILKYDHCFFIFHPNESYFIPIPVIKCMVKNILKSLLYVHTKKNICHRDVKPS
NILLDKNGIIKLNDFGDSEYMINKKIKGTRGTYKFMPPEFFVNTKCYYGEKVDIWSLGICIYALFYKVLPFSNKS
GLINLFQEIAKEEIKYHIDRNYFLLKVRKGATKKSSHDSLSNDDINFLKIFLKKKPTERCTAEEALEHKWL
>PBANKA_136210 PbTKL3
IIILNKIGGGSYAQVFRGKYKGKTVACKLFLYNTKDLSEESYCESYISTPRSTHQYIFPKIFKSMTSAESEIGDV
NHDIEDNINFEKYVNSIKSYTEVSEKNDANISEEVLTNLCTLQRNKKIKKMANLEIFRYFPTPIKYRNYEAKILY
SLQNCEHVIKLLGVCSLRQGEESLILQYCSGGSLEKYIYYDERKKINKQNKHLSRPQVVKIFQQIAKGMYEIHSN
NFFHRDIKLSNILLDENKNAFISDFGLSTYFSLNDSPSAYAIYGNIFYAAPEVLKGEGFFKESDVWSFGVSLWEA
LTKKIAYDGLSSSETFCKISSGELHLPIPNDIPKDLSDLLKSVLEYDFTKRPLFDVIARKLEHIWE
>PBANKA_080560
MNYTLISAGSDAKIYKCDFIGKEAIKKVIYRKYYRHKKIDSKIRKLRISNEIKFTKKLASLNIDVPYLYFVDTKE
KSLYFEYVNGCTINNILKSISQYQPNIPKFIGITLAKIHNGNVIHGDFTTSNLILRYSYINENEIFNNLNNAPYE
LNDIKEIKLCVIDFGLSFLSASVEDKAVDLFVLLKAIKSFHSEFPKLEEDILLGYETQSNNFDLIRKKLETVKQR
GRKRPMV
>PBANKA_041040 PbGSK3
YKLGNIIGNGSFGVVYEATCIDTSEKVAIKKVLQDPQYKNRELMIMKNLNHLNIIYLKDYYYTEAIKKNEKNVFL
NVVMEYIPQTVHKYMKYYLRNNQFLPIFLVKLYSYQLCRALGYLHSKLICHRDLKPQNLLIDPKTHTLKLCDFGS

AKSLISGQRSVSYICSRFYRAPELMLGSTNYTTHIDLWSLGCIIAEMVLGYPIFSGQSSVDQLVRIIQILGTPTE
DQMKVMNPNYADVKFPNVKPKDLKKVFPKGTPNNAINFVSQFLKYEPLKRLNAIEALADPFF
>PBANKA_070970 PbPK1
YKISHIIGNGVYGVVYKADCLENGNVVALKQTYQKSAKYFKEIEIMKKLKHPNIVKLKHAFYTLCPNGGIYVHMV
MEYGNTDLATSLYYITIKNSEHVNKTYKNINNSKYIKNDKDNDVKLKNDCTNFIASEENAKCVNNNNINSINESE
NKENLEKLQMNKCNENCMNVHDPNYNEMTKNLNKSENKCNEKNIDVNSYNNTEKEINSSNNNNVKSENDCLNTYG
NIINNFNINMLQENINNICSCHNLSDYIKKCFFNENQVKIYLYQLIRATLYLHSLCITHRDIKPQNILIFLNKPN
NNTDNSHENEKTQNKKCVVCIHNSVKFKCIMNKRNMNKSLDENNSNVNIKNSIVNNDCNNNEYVVNNSNALLNED
IEKYHKNYTSKFNSINENNDIKKVNKENGYYNCCSTPISMLSEKKNNSNIKHAREKRSMSFSNTYTFKNSDNEHF
DYMDNSNKDNFKKALKRSKTDLVISLKMNQTNTFNNEKKETLLPLSDVDKKCIDSCENVKEKNEITNFNNDELDN
IYNENNLDKIYMNSIVYKYIKLCDFNTSTKLKENYKYLSYVCSRYYRAPELLFGSNYYSHAIDTWSIGCVMGELI
LGKPLFLGDCASDQLVEIIKILGTPNDEDFLSFRSIYKNVKFPNIKPITLKKLISNNSSKESIDLLDKLLQFNPK
KRIKLCSALLHNYF
>PBANKA_144300 PbNEK-1
YEVIKKIGNGRFGEVFLVKHKRTQEFFCWKAISYRGLKEREKSQLVIEVNVMRELKHKNIVRYIDRFLNKANQKL
YILMEFCDAGDLSRNIQKCYKMFGKIEEHAIVDITRQLLHALAYCHNLKDGPNGERVLHRDLKPQNIFLSTGIRH
IGKITAQANNLNNKPIAKIGDFGLSKNIGIESMAHSCVGTPYYWSPELLLHETKSYDDKSDMWALGCIIYELCSG
KTPFHKANNFSQLISELKKGPELPIKGKSKELNFLIKNLLNLSAKERPSALQCLGYQII
>PBANKA_124070 PbNEK-2
YDVVKSIGRGSFGIVTAVKNEQGEIFVVKQLDISCMNYKEKMNVINELKALIEVSTHPFIVKYKEAFIEDGTLYV
AMDYCSKGDLKKYIKRAKEMKTIIPENKIKRWLLQIITAIKFIHEKKLIHRDLKCNNIFLDENEKAKIGDFGLAK
LLEKTDQTNTLCGTVGYMAPEVCKNVPYSFSADIWSIGVILYELIALRHPFKSKNGNMLSIVQKVCEEQPDPLPS
IYSKDLRNLCYWMMSKCSEKRPTVYDIIGTEYF
>PBANKA_061670 PbNEK-4
YEKIRDIGKGNYGNTILVRDKKNDHYVMKIINISQMSQKEKRQCLKEVELLSKLNHPFIVKYIESYIEGETLRIV
MKHCKGGDLYHYIQNKKKQNTPIKEKRILIWLTQILTALKFLHSNHILHRDMKSLNILIDSDKRVRLCDFGISKV
LENTLDYANTLIGTPYYLSPELCKDKKYSWPSDVWAIGCLIYELATFRTPFHSTKGIQQLCYNIRYAPIPDLPNI
YSKELNNIYKSMLIREPNYRVTVQQLLVSDIV
>PBANKA_060060 PbNEK-3
YKFETVLEIMTSNSEIHLVKSLDTNETFISKVYDLYGINENDLKNYMNELYIMKKLENCENIVKIVDFIKKNDSL
SFIIEFCNQGDLYSDILRRKINNEHYSESEIFNILNQILNGLSSIHKNGIIHGDLKSTNIFIKDDKIKIGDFGIS
QKGSNKNLGTLNFLSYESIKLNKTNKLSDLFQVGCILYELVTLSSPFSANNINDMIRLFEDKNYKNEIIKNISSF
YSIQLVDIISKLLSLNALDRLQVISNYNIVRN
>PBANKA_010410 PbNEK-5
FDIAGFLGDGAHGSVFLACERRTNFICVLKCISKSHLVKSTQEALLRKEIELQAHLKHPHIACMYTWFHTSSHVF
FVMEYCSNGDLFTYLNEHGPFSEKKVATMLFEIIWAIRTCHDKRIAHLDLKPENVLVNHEEKCKLADFGLSAHIG
SKHKKKGISHYRGTHDYWSPEQCARHQKKKKNFGEFDQKTDIWTLGILAFELKFGRPPFGSTNEEREDIIMNRIQ
DYHWSQLFSEKVKQDLIDKLSPEFKNFLNLCLDKNPKKRPTAEELIQHPFI
>PBANKA_083560 PbPKAcat
FNFIRTLGTGSFGRVILATYKNEDLPPVAIKRFEKSKIIKQKQVDHVFSERKILNYISHPFCVNLYGSFKDESYL
YLVLEFVIGGEFFTFLRRNKRFPNDVGCFYAAQIVLIFEYLQSLNIVYRDLKPENLLLDKDGFIKMTDFGFAKVV
NTRTYTLCGTPEYIAPEILLNAGHGKAVDWWTLGIFIYEILVGYPPFYANEPLLIYQKILEGIIYFPKFLDNNCK
HLMKKLLSHDLTKRYGNLKKGAQSVKEHPWF
>PBANKA_100820 PbPKG
LLTERIIGRGTFGIVKLVLHEPTKIRYALKCVSKKSIIELNQQNNIKLEREITAENDHPFIIRLVRTFKDSKYFY
FLTELVTGGELYDAIRKLGLLSRSQAQFYLGSIILAIEYLHERSIVYRDLKPENILLDKQGYVKLIDFGCAKKIH
GRSYTLVGTPHYMAPEVILGKGYGCTVDIWAFGVCLYEFICGPLPFGNDQEDQLEIFRDILTGQLTFPDYVTDTD
SINLIKRLLCRLPQGRIGCSINGFKDIKENSFF
>PBANKA_146000 PbPKB
FNFLKVIGKGSYGKVLLVKHTQSNKLYAMKILKKDNIISQNQFEHTKVEKNILKCVSHPFIVKMYYSFQTSKKLY
FILEYCPGGELFFHLSKLTKFTENIARFYISEIIIALQYLHKLNIIYRDLKPENVLLDKNGHIRLTDFGLSKECI
SDNNSAKSLCGTPEYLSPEIIHQTGHGKSADWWSLGVMLYEMVTGQLPFNGKSRDLLFENIKYKKIKISNRLSPE
VVDLLKKLLQKNPQKRLGSGITDAEEIKKHPFF
>PBANKA_082710
FTLKKKLGIGGFGEVWSVSLKSDIEENSSFFFFSIKKTSHFALKIMDINEFNLNESIIMREKAHINVINFYCVFK
GYQLLTNRQHEKERKESFCFLLELADTSLEKVFSDKNVAYNLNFVRLTLMEIANIMSYIHKQTPNHEFYIYRDLK
PDNILIKNKKILLTDFNLSRKIDADFEYLMSQCCGTKGHLAPEQKSLVYDQKIDIWAFSIIIAKFLKHKNFNYFS
YDNYKIDLKHFEIQDKFLINLLLVCVDESPFMRPSFSEIFRLL
>PBANKA_146050 PbGAK
IKEDNLISEGAYSFVYLANDLNTNKIYTIKKTICQNKEKLEMAKKEINILKSLPPHKNIVQYYGSTIINENNYKI
VIMLMEYCERGNLLNIFEKNKDKIKEFHIIKILKDIISGLNFLHTQEIPIIHRDIKLENILCDKNNVYKICDFCS

HTVSNSFFPNDLKKNELNLLKYEIERDTTIYYRPPELIDLYSNGEISTKVDMWMVGCVLYLLLFGFHPFQGTVST
KIIRRNDENVDINPSNNSFLSILNGSFVIPHVTKYSKRIISILLMTLDKNPQIRISSSTLLLIL
>PBANKA_113310
FSDAGNIGEGGFGVVTKMRFLSFPQYYAIKKISKDHIIKSQAAGQAYLEAKYHSVLSHVNVIKMYGCMQDDNYIY
HVLEYCPKGSIYSISKNFKKRIIPEELAYKYFCNVVNGLYYLNQMGIFHRDIKMENVLVDHKDNAKLSDFGLSAM
ILGEKSHSSLCGTLVYFSPEIINGEGYDWRSDIWSLGILLYEMLVGDVPFDGTKTQIVQSIYSCNLNFPNFINPL
AINLIKKALVVDVNKRIKLSEIASDPWM
>PBANKA_040740
IVDKHPIGNGRTGLVFKAIIRKENLQVALKVMAKDTILSLKIERQVLKEIIIQSSLKHINILDLIAYFEDKTRLF
LVLEIANGGSIRNKMKLKHDTFKEEQVALYVYQIADALSYLHNFNIIHRDLKPDNILIHYSDSDVYSDENVSKIY
KYGIIKIADFGFSCQLKNKRQKRSTFCGTVDYMPPEIINQIPYGCNADLWCLGIVIFELLVGFPPFTDNSQERIF
EQIKEFDFHFPKTVSQMARELILRLCSRSSEERISAEEVKSHPWI
>PBANKA_090380
YIVLNNLGKGTYAEVWKVKHKVTNEIFAAKLLQPNQFPKESFNRIVEMFTKEIINLSICQCPGVIKLHKVIGGKE
GWILIQDYANDGTLWKENLSNNMSEAFLYFIQLLQGMWYIQDMNIVHRDLKPTNILRYSNKKIVIADFGWSEHID
SCNLHPNEWPGTLEINPPEVLRNTGPMTEKIDNYALGMNMILFISGRFVCRQKGVECSKVAQIILKTVHNLRYSK
PPSRFRENLKAWDLFVKLTSSNPNERLSLQNVLDHPWV
>PBANKA_031420 PbCDPK1
YFKVRKLGSGAYGEVLLCKEKNGHSEKAIKVIKKSQFDKGRYSDDNKNIEKFHEEIYNEISLLKSLDHPNIIKLF
DVFEDKKYFYLVTEFYEGGELFEQIINRHKFDECDAANIMKQILSGICYLHKHNIVHRDIKPENILLENKNSLLN
IKIVDFGLSSFFSKDYKLRDRLGTAYYIAPEVLKKKYNEKCDVWSCGVIMYILLCGYPPFGGQNDQDIIKKVEKG
KYYFDFNDWKNISDEAKELIKLMLTYDYNKRCTAEEALNSRWI
>PBANKA_040820 PbCDPK3
YNLSEEPLGKGTYGCVYKATDKLLKIQRAVKVVSKKKLKNIPRFRQEIDIMKNLDHPNVIKLLETFEDEEQIYLI
MDLCTGGELFDKIIKKGSFVEMYASFIMKQIFSVLNYLHIRNICHRDIKPENFLFYDKSTESLIKIIDFGLAAYF
NDIDYEMKTKAGTPYYVAPQVLTGCYDYKCDLWSAGVLFYIILCGYPPFYGESDHEILSMVKKGKYNFKGKEWNN
ISEEAKDLIKRCLTIDSGKRINASEALKHPWF
>PBANKA_061520 PbCDPK4
YKGIKILGKGSFGEVILSKDKHTGHEYAIKVISKKHVKRKTDKQSLLREVELLKMLDHINIMKLYEFFEDNNYYY
LVSDVYSGGELFDEIISRKRFYEVDAARIIKQVLSGITYMHKNNVVHRDLKPENILLETKNKEDMIIKIIDFGLS
THFEYSKKMKDKIGTAYYIAPDVLHGTYDEKCDIWSCGVILYILLSGCPPFNGSNEYDILKKVETGKYTFDLPQF
KKISDKAKDLIKKMLMYTSAVRISARDALEHEWI
>PBANKA_135150 PbCDPK5
YELDKYKLGKGSYGNVVKAINKKTGQAKAIKIIDKKRINNIERLKREILIMKQMDHPNIIKLYEVYEDNEKLYLV
LELCTGGELFDKIVKHGSFSEYETYKIMKQIFSALAYCHSKNIIHRDLKPENILYVDSSDDSPIQIIDWGFASKC
MNNHNLKSVVGTPYYIAPEILKGKYDKKCDIWSSGVIMYILLCGYPPFNGKNNDDILKKVKKGEFVFDSNYWSKI
SLDAKELICECLNYNYKERIDVHKIVNHKWF
>PBANKA_092550 PbCDPK6
LSFKKILGCGAFGEVHLVEDNICKLYKVVKILKKKKMKNIKVNEEINVLIYLDHPNIIKIFDVYESVNCTYIVME
LCEGGELMNKIKKPQIFSETYIKNIMFQILCAIAYMHSNNIAHKDLKPENILFKTDGYDTLKIIDFGLAELINKS
EGISKTAAGTVLYMAPEVFKKKFTIKCDIWSAGVIMYFLFTKSLPFTGNTYEEVKQNIFNSEPDYQFLKLKMSKP
ALHLLKLMLEKDYSRRPMAAVLLHHPWF
>PBANKA_101980 PbCDLK
YKFGKILGCGSFGVVRECTNKKTNEIYAVKIIKKKKKHKKNSNFEKMVKNEINYLSIMSHENIIKLKDFFEDKNK
FYIVLEKCEGGELFYKIVKNKCLLESESIQIVRQVCCTLEYLHSNNIIHRDIKAENFLFKNKNTENIKLIDFGMA
KTVNCEYLTELCGSPHYISPELIRKKYTMSSDIWALGVMVFFMLTGKYPFEGKNTQKIVDEILNKNINWKDNEFS
SLSVEAIDFLKKLLERNERKRLTAFEALNHPWI
>PBANKA_131370
YKIINKIGDGNFSKVFCCRGENKKKCAMKLMCCPLKKTSHYNCFKRELFIMKTINNKHPYIVKILDYHEKIWKKY
YIVKLILEYCEGGNLFEYIKINGSCTHSEARVIIIKLAKTIQYINSLKIMHRDIKPENILLRTKDNIKSVVLSDF
GLAKITPSNQSVVKSRSVCGSDFYLAPEIIKNKEYGIKIDIWSLGVLIFFIITGKVPFTGKNANELYNNILKANI
PELLSKEKSLNIQPGLKNLLENILVHDPEKRFSCSDILNHRWI
>PBANKA_145340 PbPK2
YVLNKKIGKGSFSNVYIGTNILYGNRVVVKEVDKSKVKESNVYTEIEVLRKIMHKYIIKLISAYEQEGYVYLVLE
YLKGGELFEYINNNGPYSEQLAKKAMKRVLIALEALHSNGVVHRDLKMENLMLENVNDPSSLKIIDFGLASFLNS
PSMSMRCGSPGYVAPEILRYSSYGTKVDIFSLGVILYNILCGYPPFRGNNVKEIFKKNMRCHISFNTKHWLTKSE
NVKEIILWMCSKNPDDRCTAIQALGHPWF
>PBANKA_092520 PbCDPK7
YEIHEQLGQGKFSKVYRGINKQTNSEFAIKVINNRSVSIYEKELLRSEISILRLLRHPNVIFLKEIINTKETLYI
SMELVKGGELYDFLLSETRLSEIHANKIISQLIKTVAYLHKCGIIHRDIKPENILLTDKSKDAQIKLTDFGLSTL

CAPNELLKEPCGTLAYVAPEVITLQGYNHKVDAWSIGVILYLLLSGKLPFPINKNTEMNIQKTYVLSFRDNIWKT
ISSSAKDLISKLLELNADKRISASEALEHIWI
>PBANKA_040940
YKLLKVLQSAIYGTVYLSEIIEDVNKKLINTKKAIKILSKHLIEMAKERIQEDPLSEYYYRDSMSGHSNILICDS
IFDDAAYIYMVMPFAMHGDLFEVMKNRNKAFSEEESRYLFYQILLAIKFLHSKEMALRDISLENVLLFENETNGL
IYPVLNDPGQALHFNVNNKNEVILEEYTKTFGKIFRPPEIHAKCKYDPTKVDVFCVGYILYFCLTKQELFRSSVE
KDIYWNMLVHKNYRELLTDKNGIHLSNDAVDLIFHCLDPNFQTRYSINQVLSHTWF
>PBANKA_040110 PbSRPK
YRIEGKLGWGHFSTVWIATDLKSKPLKFVAIKIQKGSETYTESAKCEINYLKTVKINSFDSSWVEFKEQQRERLF
HYNMTKGVVSFIDSFEHKGPNGTHVCMVFEYMGPNLLSLIKHYDYKGIPINLVRKIATHVLIGLQYLHDVCKIIH
SDIKPENVVVSTLTNIPKPRDYTKSKLANNNDDTNIKNVTKNHCTSPSRPEQENNINTEKDFDKVEPQIDETKQG
ETEQDDTESNATAVKEQNEFDNVDWNKLTKNEKKKLKRKKKKMFKKERLKMNEANNTEKNQQTNNEAKINNLSHA
EDNRQFKKEDNENFRNIECIKTDDISLEQIQISTKMTPNNSMNANPKVNINTNKDNSTISGDCNNQNLNENSDMK
LCKDETKMDSKQIISNTVGKKLSFVNANMDNFSINENKLSDLSKEETKENTKCSIIEDNEKNKIDDNNNNLNIKK
KKKKINEPPYVKHRLKPSNSDPSLLTTYYNIHAIQETLTRKPYHYNNYFLNNPEKFGYDKSPQLLRRLPIDYLRN
DSSLEASNNSCNDNNENTNDTYSEKSEIYVDKDANKFPIYCGMFNHLIHPEAMKLHESNKKKNIYSEVPNENQSL
GNNKNTNKVVYIKTEEGDYCIRPYDPTVYYHEKSCYKICDLGNSLWIDESRYAEIQTRQYRAPEVILKSGFNETA
DIWSFACMVFELVTGDFLFNPQKSDIYDKNEEHLSFIIEVLGNIPKSMIDSGYNSHKYFNKNTYKLKNIKNIKRY
GLYKILKYKYGLPEKEINPLCSFLLPMLSIDPQTRPSAYTMLQHPWL
>PBANKA_093300 PbPRK4
SVVCELVGKGVFSNVLKCYDMTNKIHVAIKVIRDNHMMHKAAEKEISILKKLNDYDKDNKKHIIRLLRSVKYKNH
LCLIFEWMWGNLRIALKKYGNGHGLNAAAVHCYTKQLFIALRHMRKCRIMHADLKPDNILINEKFNALKVCDLGS
ASDISENEITSYLVSRFYRAPEIILGFRYDSQIDVWSAAATVFELATGKILFPGKSNNHMIKLMMEYKGKFSHKM
IKGGQFYSQHFNDNLDFIYVDRDHYTKKEVVRIISDLRPTKNITCDLLEHQYWLKGNSPKMQFLKKKIKQLGDLL
EKCLMLDPSKRYTPDQALQHPYL
>PBANKA_030850 PbTKL1
IWLLKLIGRGGYNNVYKCLYINNIKKCRNYKMSNYRNFNINHSINNIALKICIDKKYSYDFFSELKILSILRHPN
VSLFLGGIRDPQAIALEYIPYGSIFDILHKKKTKIKILDIIKMCKDITSFMSFLHNKGILHCDLKSPNILLSESG
EIKICDFGLSIQNFDNKPKYLGIVGTYQWTAPEILRGEGYTKKADIYSFGVILWELLHRTIPFNDLKHPLDIIAQ
VGYLNKQLIINNNINNKLLYVLTSCLHKDKRKRKSFFFWSEYF
>PBANKA_112690 PbPK4
FQNISLIGQGGFGSVYKVSHRLEPGSPTYAVKFIYLKVSSLDNVNSRRYFREIAANRDIYSKHVVRYYTWWCEEP
QFLPMDIIPKEIQNTLKKNKDPFKKVCNKNKKDNDYSSDCTVSSGENNKFDLKNYKKVITKKNSPKLKFYSDNDT
PYNKRKNINQKNSFLNDKNLSDNIYIIENNKKKKKKKKKKKKIIYKEKKKGNIGINEDNKYSTFYEQNNPNNFSS
TLQEYDPFGYGYLSENERDLIVFADNDESNGSGHSKKNDNDERKSLNNQNGIYNTGGDISKNGNVIHDDSNMLAC
QQSDKNSMTIKNTQGTSINGTINRNTISDETGTQGTNNNPKYSIDYHIDAIVKPKGESFTWVEKSPSNKYKKDSL
DIINRNRKLIEEKNKKEKGQEKEKYKLKMNGELEKKENANKIKYYKKKNVGPEFSIVLLLQMEFCKGFTLRRWLD
RSSRSDKPLYFTYGDKNTNHPLEFDLFKQLIKGLKDIHSTCFIHRDLKPENIFVDLDTYILKIGDLGLVRFIEEK
KRENDLNNIDNFKDNIYTEINHNTITSQISLKGQMIGTPGYTAPEGGALCDEKADIYSAALILLELLCPRFNTIM
ERYKTLNDFRNYYTVPDYVKIHLNPWYILMLQMSKPNPADRPSAADLYNKI
>PBANKA_130840 PbeIK1
FIFMYLIAAGGFSCVYKIKKKKSNKFYALKKIKFSANESNYEKKVLLNLREIECLRKLKNHPNIVSMNDFWLEVV
QTLSKSKRERRGRRKEQQREQMGDKRREKRQQQRREKRKEQNTNTKKRVLITLSDHKKKKLKHLSCPENALNISN
ITNNERNVLKKDNWKNLILLKNFKKEKHNYNFNHQIELNKYNIMCLWKILNQMCVCKNEKKNIESLLPEQLIKNF
KNFLFEKYILAIYDDCSYLNNKNNKTFIFFNNNLGNILHYLWWSYLGKNGKEKKNDIFKLLKYVSDNIIKDNTNL
YNIILEFRHSLIELSRFPSNELGNVILNMRIPPNGSCELSEYISNMAKINKLEIYRNKNTLEKFIFKINCNNFDV
YKMWINFKNDIIYEGKDVYKEHRKINLKRKIIKKKDIWIKGKKEKHLKNTIGNKCIKKINIYYEKPIHVFVYKSL
TYKRQKHHKLWRKHYNNKKNWKYCLNKHENSKKYILFSKICRLMKTQMNKFKREEKFEKKKKIAITNIKVNYNDF
EQDISSFNIQIYNKKNKNQLINRIKEQYEQLSISLNPYKLTYENENILRYNEHNYLFGLKNDNEKENIYNAIYFL
NFYIWIRREINEYAIISKKRQICQNSRNYQSKKKFYIKRHNQKTYFFENIIFYHYIIMLFLDIEKYKNKFVSLFQ
YNLYRKLLKISKRIVLMLHRIETNVICIFLLKHFEDYFIRKGIHIVQDKSDRSNKGDEIGIHKMVKIWKSMIAIS
LIFGKKMYKKKKNIFNFFIELFLNNIQINIFKKFEILYLIIYFYNYFEKSKQFDIEGIGDIIYVWLSLINLFYDD
KGKCIKILSKIFAKLNKKLYYVYWGKLYIIMNWTTIVDTIFIRNVLSINREGNYYWVIIVLKMINYFVNVAYTLT
RMDIFFIKVMIKFYTRIGSAAATNSVSKNSYNEIFNNIFTLNFMNYIIYNSYFENEKKNYDIYTKYAILFIYCFI
IQAYYFDTLFNIRSLESNEIANNLFPGYNYSYKNILLFYERLGRVIKNSNNTKICKYMWRKFINMWSNSIVIKEN
IISCLTTSRHIYFNILMNFMKIYCLDNILHIKKKKKKMNTPIVLTSKNDLKKWKDCELTKNPKSVKKGILIKKKN
RNNNKKYKKKLKETHFIYNIKKVLFKKLVNINIETILYEQNGQCYILVFGSVIKKKNGQIKVKDTKIVRDINIIR
DYRIYFYNYLEYFYKNNAHISDNINLIYARKWPYNNKNAVKQFCPYFDKIKDGNRKDIVSLYGNKILVKQNFSKI
GNKIKNKKNNLCKKKMRTQKSIYNSNYWREKKENKLLNGNVNIIKKYKSEKIKKKELENFFDNIVYSSENDDFKI
IFENATNSNACSTVDLASPNELNTKRNNINKKLKFFKHKKNSKKQKNYKNHKSHKKIFFKSVNNANRFFVTNVEP
NPIMSNNHQIADSNDIYNNFSIQKKYEYNHKNCGNIYNTKDCDENDSSYICFLINEHEGQVLSHRHKELYKNMLG

MERGNILYRDNACKLKDDFSCLHDQCDNSMIKACGTNELIKESTKIKRENMDKINKMNEVNQHISLETLKYKCSF
KKIDKNLIQNKKKIIIRKICQINKTFYFKYNKINDKKRIYFDSVLCKSERHKTYKKRNENIKVILLKTLKGESNE
YVLTTYLTEIYSDNINDPFENSRKKINSNEIFYQKTFDMYCIEDEGEVYEEQKRVNKNNKKKKYINEMIKMDTIR
TDFEDNFTNSYNKKCNILKMASNINNKNNSGKKERDIKRQFLITNKKKHENNIKIFYNLFKLEESNVNSNPQTNP
YYETVIHDNEDNIFYCLYKYIQQQVYRYCNCDIDEYTSNCIDKNVNKWEWYGFIKENENYDKIKTEINSNSFYNC
REKHNICNSYNSVYQLEFRKLGNASKEKNFEEKKKIIKIEFRKSFNNFKLPSLLCILKWDNFFKPHFIIRCDNFL
HTYNIYFDFFILLMNLFHKGEGSNLYRFNSNKSIIYNPYLSHQIYMVTKYFISNVHKINNKLPIHLENDILEIYS
YNRFLTIPNKCSFKNCGNDNNNYDQRSKKHYFTKCGILNTEKVKPSKKRRIGWDGQRQRKRKDIINTLNEENQNM
FCKNKEKKEENYKKIDTNISQFSEKNPVSNIDNEKNKQNFIKNKKYKFNLYIRMEYCKDTIENYINRRTRINIKR
NIEIINMIIMGLNYIHNNNIMHRDLKPSNIFISNNDIVKIGDFGLASYDYLDDHKINTTKEEEIQKDLIINKNCD
KIFFCNKKKLFSNYNSVFPLENGQISDVHNTKGDYNESSISKSKKFAIQNKNRNLRSCKRIFQWWSTIGELNILS
KNRRRLTKFKSGSNTIHIRKSTLDENIIVRHANKCHNLSFSQNREHIDRNRMKKCNIIKNHIIKSNKSEKMNISM
NVFLRCTKTRRYFTDEDKSVETRKKCSKTSEEENGNICDTKKKKNDIGEKMDKNKIAAQKKKKKKENKHPIGRRS
TNSSISSAIVVKRNAYCRLEIEKYFLSKSFQNCRSNKKKKYINIKTIKNKFCSASNKNFGAKWMRIYRKGLHHDD
IQEKSADQTTEQMGGCNKTVASDFSSNLKNKKESINHTLGIGTKLYSAPEQLEGNKYTKSVDIFSLGLIIIDLFI
KTETNMERTQILCNARERILPDLLIKKHPNVASLCKKMLSLDYKSRPTSAQLYNKI
>PBANKA_020580 PbeIK2
FSEESVLGCGGFGYVMKVKNKKFNIAYAVKKITLSSYCPNKRGNDNELVNKYNNHIKKNSKHNDIEPMISGSFSS
GNNIMNENNSLIMEEVIMIAKLQHENIVRYYDAWVENNIDYYLFKEIENNYKYVIKKKNKGNKKNIYYIDEISNI
RNYYNKKNKENIKINEKYLYILMEYCPGKTLREAIDCGFIYKNEKLIWELIKQILKGIYYIHDMKMMHRDIKPSN
IFLQINDDILSAKIGDFGLTTKIDNTQINPSAGTVNYMSPEQLNGEHFDQKADIFSLGVVFFEMFHEPFSTSMER
SIVLSNLLKCIYPESIRSDNKIFQFLLSLLEIDPQNRLSAYSLLHENFF
>PBANKA_141360 PbPK9
YIVEKRIGSGGFGIVFQGIHIQTKQKVALKFIPKSNFLDVTDVHRVFIEIQTLRGLIHKNIIKMYDVNHFQNYVC
LIMEYAVNSDLKKYILKNNGYLSEKETYFLFLQIVKGVYYCHSKHIVHRDLKLENILLDENMTCKIADFGLSDFV
NVDQNIKTEAGTKLYIAPEIIFNQTTNYSVFKLDIWSLGILLFIMTQGYPPFIDVGKDIKYFEQSTLKYSNDISD
DLKDLISLMLNVDPNKRPIIVEILNHRWF
>PBANKA_131800 PbKIN
YIIIKNIGKGTFGKVCLGMHTYTYEIVAIKILNKKKLLRIISYDKILREIKIHKKIDHNHICRFYEVHENKNNIY
MILEYLGNGDLLTYICKNSNINENIAKRILYQLISAIEYLHKINIVHRDLKPENILLDHNNNIKLIDFGLSTIYS
KNNFLQTSCGSPFYTPPEILLGKKYNPELTDVWSLGIILFLLLNKKLPFNHNDINKLFQQIIKGLMQFEPHVSIN
AKNLIQNMLNVNCKNRYNLNQIKNHIWF
>PBANKA_130920 PbLAMMER
YKVMRKMGDGTFGRVLLCQHIVNKKYYAVKVVRNVKKYTKSAKIEADILKKIQSNDIKNNNIVRYHGKFMYHDHM
CLIFEPLGPSLYEIITKNDYNGFHIEDIKLYCIEILKALHYLRKLKLTHTDLKPENILLDDPHFEKKIVTVKRVT
DGKKIQIYRSKSKGIKIIDFGCATFKTDYHGSIINTRQYRAPEVILNLGWDVSSDMWSFGCILAELYTGNLLFKT
HEHLEHLALMENIIEPIPKKMLYEATKTNGYKYIDKNDLKLAWPENASSIDSIKHVKKSLPLYKIIKHDLFCDFL
YTILRIDPTLRASPVDLLKHEFL
>PBANKA_090110
IRMMNLIGRGGFAEVWEVFDSINLEMYAAKIHKIEPSMTNEIKNKIIQRAENEINIHIHCHRHIFIVKLEFFFVF
GSATNLLVGMELCDVDLDKYIKYHGPINELLALSWIKQILLGLLYMKNLPTGKVHHCDLKPANLLIKDGIIKISD
FGLAKLILPDTYQYYNGGGTLYYQPPECLKPKRNLLITDKIDIWSLGCILYEMIFCERPFQFNYLEKCSKELLVN
KMKRGLSYPKINQHISKITLNYIEYLLNFDHECRPSIEEALSYPIF
>PBANKA_112270 PbTLK5
IKIQSYLSKGGFGVVYKGILLKKVNKYEEICDQNSKYTSDFHNGLDDGNSVEKEYNNIIEKEEICDQDVKIKRKK
MLINKQKENNDNKIIAIQDDNCNNENVKGQKNEREKEIHNKDFENPNSEQNNFRRLYAEVMKVNNKNSNHCGNNT
NNSSNNNTNYSRCGYQKNCQNYNLNCNENHDKIQECIEQMDFLRNKMIEKIFETFNNQENVFSCNNNEVNDNNQN
KKEYNMRINLKEDNKSVIENILKKKKIINEYNNGNDKNGVNYSEQANKDINEIFGEQINEDNRYSINSNGENKFK
LLNNFPFFGNKMEKKKKNYDIEEELKNLTNFGDNMYQNNEHVKKNEQYSHECIHKGNVLPQQCLHSEEIKELKKS
MLNFIYGNKKCDDIVIEKNCFINEAEKIINKLQSINKDLNKKYNDEIISYLLVEIYHNNIVYNNNLSFLCIKSKN
IITFSLNKDYLYIFDSQNIFHYYSDKSIFKIVFIDDYKILNKYKHFTVFNILNNDKVLKNSSILKREINKHKNGL
NKLSIIPFSKLNMNHLVNALIFGFLKFIFNLRKKYHTIKKDCYCSLCKNPKNTNFMNNIASFSDRGSNPNDYDQN
FDDDLESILLQNKINILDELICINSNNNDENIFSYPKNKNTTFDRNSNLNNHPPEESINDDNNAHIIDKEKKTNF
RGNDNSIYHIVNTSDSGTKNNISNVNINIKNNLNKPNIAQNDEVQLNGNKGKIINSSNQGLGDSTLLNSNNTDEC
KTTIILNLSKNKNVSKDYINEKEYNNYVSKIKNNEKNKEEEEDDEGNTSNKMKNHDKKTYIQKEKLKINCNSENK
KNREGNDFETPDNEEREVNEEEENGNKDGEVYMQIPVAIKIHDLKDSKNLKNFLREIEIYKNLQRSNICKFYGIC
IKHNKLMLLLEYYAKGNLFNFLKNKNKIHKKQRLEWAIQMCSIVHELHSHNPPIINGDIKTSNILINNNMDLVMC
DFGKARFKNSKLYSNFGSYRYMAPETFSCTSEVTEKIDIWSLACCIVEIFCSKYPYYNFSKNTKIRHELIVNKRT
PHIPSFLPNSIKKCLQKCFSFNPEERPSAYEMYKAL
>PBANKA_142160

NKKDRTLDNNGLYKKNIIQLVVKIKHKELYSKINECNKITEVEIHKKLKHCNILNMILSAEDEKYICVFLEYSSI
GDLYSFVGFNILKEKEVKIIVSQILFALYYLHIKGIIHCDLKLENLLLFNFDEESFLFESNLLSDIKMCHISQFK
KLNAKKLVHSIKENIKNNPNIKVFKYIVKLCDFGLSVKCEFDKFYPFNGIRGSYGFIAPELFQECSFNNKIDMWA
LGIISFLLLGGYKPFYPCSRFEEKVIFHERYWHNISPEAKNFIQSLLEIDPFKRMNVIEAMDHPW
>PBANKA_130520
YIILEKINTGSVGQVHLALDKKQVIFIVFSKAIDKSTVQGDEGLFQKLKDEIIVSCRMNHPCVVKTINILETRDK
IIQIMEYCDGGDLISYVRNVLYLEELSAQYFFRKILDGLKYMHSNKIAHRDLKPENIFLCKKVLNQKEKTLIRGK
LPSCFEYELKIGDFGACCFNDASNKLHYDIVGTLSYAAPEVLGCDKNNGYDSEKADIWSLGIILYAMLFGLLPFD
NEEKDIKEAHNEIVNNKIIFPKNRINRCSNNVKNLLIGMLNINPINRLSLDQVINDPWV
>PBANKA_080220
LIKDNTINFLYKCYDIKNNKNVVIKCVNKEKQLSIMSYNTYTNIYKIIKKIDNENIIKIYDVLENQSHFFIIMEL
CEGTDLVDYVSNENISFEKIKDIIFQLLSGISALHDNFIIHRDIKLDNLMFKDKNFEKLVIIDFDMSIYICAKDQ
IYPSKSDCIYTSETKNTIYDNKFDENYMNMCNSDQLFSKKIGISGNFNTEFSEKSKNNICKEENNAATSNTFTDY
ANQGKHENPNKMENHDSMNIKNSCLYKNEPNMNTLDSNNTYTNKVTENDRKCSNLNSWHFSNSYDYIKNKNKTHN
KKSTSLDYNYIRKIENNFLSTNTRDIQKPFINNEDKIIYNDLIIGTKEYMSPHCLKGMYSTKTDIYSVGVTIFLI
IFKSFPYLFEEISIDKWEDEIINKNKEIVIPFSFLFHNVNCTYFIKLMDLHLINNNIYFCNDSCLLDDNLKEIAK
IRGIKFDFDKIKINQKNIYLVEILRKALSLDVCDQYSNVSQIMESSLF
>PBANKA_082690
SLKYTVIEEGSFGVVYKGWYKNMHVAVKVPVEKMVKQDPYGLTKRSINEWKILSKCEHPNIIKLHGGIIHSYFDI
WLVTKLINGSDLHTIKNNMNKEEKVLSVDISLKMCRQLAAVINFLHTPIKNKKNVIIHRDIKPENLIIDSDWNIH
LCDFGDSEETEDGNVSNVSGATWIYAPPELLTCHPLKQSSDYNFLDYAQLSYKWDIWSMGCVFQEMMNLPSPFQH
YIISFDESDQIYEKLVDVFTKKLPPCIHSKIDNSPFADIIKLCLNYDPNLRPTAKEIVDLL
>PBANKA_031140
YYIIGLIYYGYNSQVYKCINIKDKKTYAMKVVLKERENYEMVKNELNIDKFMKKYMFLKKNPHKNIIPIYDIFSD
NNYNFIIMEFCKGSTLLDYFMSLVPGSLHIYEIKKIMKNIFLALDFLHSRGLIHRDIKLENIMFTKKKMRNFNYK
QCDNFDLDNYTDPFFLFEEKGKTCNRGIVRNSVLKKCESKIDLNNKQDNDLFLLKEEIDTIHKVDVDKCWNSNGN
IEYEIDSDTSSSDNIEWNNIWCDDRYQDESSYNCELIEEFNFENSSNNISEIRKKPRSFDAYNNLFGNLDAYNCD
DSSNIENNSLDNVMGRNLVNILDNSYNFKLGKIYKNGSTIQIINKNNFQKKNIIIKKNSSLTAIKSKRSEEKYLI
NSEKVKYRDTENRNNCRKNNNDKKSSFKKRRKYFPCNLYDNLCLIDMDMMEDISNNNPNKNKKQNIICGTASYMS
PESFDGILSASNDIWACGVILYALMDGRFPYEIYNTMPNYLKKKILRYTKPNFDPFIWQESPDLLDLCLRLLDPN
PLTRIQNAKEALIHCCF
>PBANKA_092700 PbTKL2
FSEENKIAKGGNGIVYKGILKNCINVAIKVLKKNENNGFENELIIMSRYRHNNILSLLGYAMNTNYFYLIYEYVS
LGDLRTLLFNHYYFYNSQNKENIDFNNLSNMKLNNYNNIPKNKYITSLNNYTNLNYSKTSNLSENQTPLFLSFST
RINILVQIINVLCYLHTSSPIVYHRDLKSANILIDEKFNAKLGDFGLSFIYINNNNIFNLTGGTPGYADPYYIST
HEINEQTEIYSFGALILEMLVSKSPAIHVGKNYSCIYTRNEKCPIHCHRDKNDNDDNVFDYLINHINTNDYKSIY
SILDYSVNFPDFLVEKLTKLSFLCLNPNIKNRPSSKLVNLIL
>PBANKA_092640
FEIYMHIGSGNFSDVFMVKLKNDPSKIYSLKIFSKEKVNRMNKVNSVLTEKKVMIKLNTPGHLNVIKLISTFKDK
ENVYLLYEYADYELWEFLKTRSIGVSENITFNIILQMVHALAYIHDKDVIHRDVKCENFLINKDGTIKLIDFGTS
KDLDHIPMENNKNECTIKDELSKFSLKKKNNNINKNLKNESEENTSMLRDDESNNYVLDNDSNEELDKISNNDIS
KNCRLKEPQYNESFKYDDKNTIKCNNDNLNNLDDTIHLININKRNYRRKKTFDNYVGTANFMPPEALINKCSGKV
RDFWSLGCTIYQLVTCTIPFDGSTEWFIYNKIKKREINYPPIIPLDLVDLIEKLIVINPECRLGFKNGCEDILQH
PYF
>PBANKA_094200
AVLLNVIDKQNNTEKIIKIINKKKTLTAFGETWENMIEYILSINQHKNLMKLYDIYDDGKNFYLIMEKLRGKELF
TFLVYKKKVKENVCKYILMQILQAVNYLHYHNIIHRDIKPENLMFRNQKRKDKNYEYNYELVLIDYDTCQFIKPQ
TRFNLFPTSIYNCKHVNLTNIKTIQNSKDINNAFLNKSKHIKLVGTYGYIAPEIIKGLNYSISSDIWSIGIILYI
LITGVTPLPMCFMINYRNTKDILIKKEKKGINFNLLSFNNYPLAKDLCQQFLQFDPKKRIKNTIIASYHPWL
>PBANKA_130690
YKIIKVLSKTTFSTTLKCLNLDYKNSIKNGEDSSNLLTKEKVSELESVCKKSKILSDNPNMSFTQNLKDLNNKYP
FDISSIKKNNSSEYIRNSTNNKNNGIKKKYYKYVCLKVMKKGKNYFDQGLFELTILNMLSEESINQTSMGNNKSN
DITGSNILTNKNIIQLYDYFYLKGHLIIVTEYMESDLYNYFIKKGKLGTLGQLQILTKNLLQGLAYIHSKKLIHC
DLKPENIMINIKKKKKKKKYPQHPTGNNNDETSNVQISHDSNEKNKYFSNDNDFDKNRENIFSSDQFSKIKIIDF
NSSIFEFDKLEMYVQTRPYRSPEVLLQHNYDSKIDMWSLGCILFEFLTKKILFDHKNIYRFIYSIASYIGSFPFY
MIITCKIPYIFTKHGYMILKKIIYYSNDDDYPKEDPIDEIEDEPVIFNKNDFFCLNKKCHQNDLLKIKNQNPKFV
HKQTNHKVYYDICYSSNTPLENNFQIDDLLFINFLLSLLQIDPCKRLNSKEALDHPWL
>PBANKA_082960
YYNGKIMTFYKYGGNSLMQWDKETKRFQYKKKVYTVNEKKKKYIIFDKKMAENKKEYYEYDKIWNPRINNDNNDN
ISNKRTKREKTYVYPEYMIAEILRQLLKACFYLYENQIYHSDIKPSNIVAKNIKKKNLNKIIFCKKEKKWYIKKY

GKIKKNKILIKIIDFEYSQKCYGEEANVGGTTSLFKPLENFRKKKINIFSKMVWIIGITIFILSTGTHPFSSINN
DMHVLFLIQNKNFDIKNSFSKYSYFSHSFKDLLQKMLKVEYTQRISFFDLFFHPFV
>PBANKA_122880 PbTKL6
LEIMEFISEGSFGAIYKAMWNNQIIALKKFNSSMTLEGMRSIAREINAYRSISHKYIVKYYGVCIDSDFIGIILE
YFSKGKIFDTLHKGEFNLTYELRLRMCTQLAEVMNYLHEDKKLVHRDLKTSNILFDDEYNIKVCDFGKTMKLSDN
GTVILEDNGGSIGYMAPECFIEGNTITEKSDIWGLSCCFIEIFFNQVPFQNIKEKEDIVVEILVNKKKPNIPKWF
NPEFTEIIKRSFSTNPSKRPSCKEYLNLL
>PBANKA_122500 PbFIKK
FICIPTLGFSKSSNRVQQMYKAIIPSKDGNSKNDVKLFIKKIPIYIWVKQFNLMTEFDGEYVTDGENFVMEAASL
AFLSEYHPRIAPKLHKILYEVDNTNLDGSNNNIPPESMFSNLTVFNNVLEERLKNNINGNIVLVSEFFSEDILDF
IDRRQKNFNMKISNNEKSYILYQCLKLLIRLHDAGLSHLDLTPENILISDNYEMRLCDLSKSTPIYTYNLRHIKD
VNRLYLFESCEPTIAKGAYMPPECWKIYWKYDTMKIKNPLRDLKSITDQEKRKQFYFDVANADKFMLGVFFFWIW
TNGNLWKCSDPLQDEDFFYFVKCDMNFDKFELTRKWPSELKSIIKGLLHAETRKKLNLKDMIMHPWW
>PBANKA_121710
MRILYSSKEKYTKYFHRGYLIKSDTNVNDTTKLYPCVFLKSGEKRLVKKIDKNIFKGEILNALLKLRTYTYKNDS
SLPKYLLKTYNIYEDDKFSYVIFENCAGGFFFDILKDNNVINENILAEWFYQILTALNFLEKTNIYHGNLNGYCI
HFKDKTRKEIRVSLLSINNNYDNIDEKGDLYGLYFIRSPQEIKKLYHDKNNSWYVGMLLYFILHGSYPFINNNVL
VNYYNIVQNNIPFTALKLDHKNYTNLMYDFLKSALEKDYDMRPSVEGLLNHPWI
>PBANKA_094010
RVFRGRYMGKDVAIKVLVGNIKNFTKFHKVLYKLYILRHTNIALIMGVSISYPFVFIIYEYIKNLCLFSYLHCVK
YKHIYVSKLLKYYQKKFINQNFQQQNNTMSSDRKYISNDDNEKINFDSRNILRNKLLEIKCKNNAKNKITEKNNL
KDEQIYSSSTSIKSLDTSSSNMNNTKLKNINFNKNRYINKIHSMFRNKNNILCGNYYYLFRKKKNNISISHEHKN
SDRTIFTNESQNLLKNKISQKKINKKLNFKAKIKINRPYAFPPLQEDFNFYLEKKKKKKKILFSYLKTHSYFKSK
KCDSRKNKLSDHQIMKIIMDITLACSYLEKQKVRWINLKPTNILLDGSLNAKISDFGIKEIEQCLDINIDYSYIV
FPNNVIKFNNKHFKNKIKKIKIVNKGSEDMLHVFSSKNHIYKYNTREINVSSNTHNSSVFFWTSPEILKGKQSPS
LYSDVYAYGIILWELMTNSVPFNYRFKSHLLASVGYAKESLPFQNIPPFIKNLIKSCINRNKYKRPTFDRILIEI
>PBANKA_082680
YKNVHKYMKQISRRICFIKEHFIMSNLRHPNIVEYIGNIITWKKNKKNKEIRENFGLVFEYIKGKRLYDILYNKK
KKGNNKENEKKYTHKNDSKDNLYNGYKQWKLNNKFILKVLYKICVTLHYLHEKNIYHGNINSKNLFITKNGNVKI
CNFKNAHIQNYYDYKTDIQNRSECSEKKNNIYYNLKANNFIPFPYITYINYTKQDTKYIDEITSTPFFLYTNIFN
LKLMNDHRNVYTAPEVLRGEEYTNKSDIYSFGIIMYELIFETLPFKNKYNNNFNFFLSILISTCYFQNYIYFDIN
QLINKINNNNIMFHIFINITILIKSCLNPDPSNRPTSKYLYYYF
>PBANKA_134590
YNKNRVGKTKHNENKKGYRSYLYNIKVIECDETIVGGHEFIFSEDNSNNIKINKLIKEGKHIKNSIYEYKEYCKD
MDNYEENKEPYFEEEKENNLNSKHRKGYKGKNKISINKSKNNNKDYEVTYETLGVVPYSDIIVLNKKELDKQNKQ
VAGFVTPFMLNGNIKKMIENINPYNKYKFNDEMIFKSLCNIIKVMNYLEEKNIVHGNIKPNNLFISNNGFNILIG
NFVPKIKLSNYYFYVINKTRTRMKYISPELLLYIKRKTNQLKKNINKNKIVSIENYHLIKNDIFCLGLSFYYIVS
MNEDILNYIDDYRVFQYKVDNIRDYIQKPELFFLLKNLLIYDHMYRPNWSDLSNLI
>PBANKA_062150
KSSEFLLGVQKYSLCEKGLNEKCGKKDKIIKNGEYNAKNYVKQNEYNMIASKFEKIKKLNHPNICRYININRKNN
DYYIFSEYYSLSLYDILNGENKNIAHFKCLRKIFGIKSQENNTKPSKMCVNMNEKLDGQTKIINHIVLKKIIYEI
LKGVEYLHSKNIHFLNITPYNILITSKGKIKLHNYCISYLFDNYEYNSKKKNKEFFSKKLFLDHIMSMENRMIKE
SKLSSVRNIQYPKYLFDLSENCEERILNSPLASLNDKKIERKKKKKKKYIFIENYFKYYNFSEDMLYFGPFFIFL
NLFENQKIKISYDLYKHIDIFSVGIVIIQIINGLIDFQFIIDIFFSNFFCSKVSEVINSEQTEFTKINNQSCDSD
FEQNKKTDRYIRKYKKINKIYESFKVVKNILQQRMSEKNMNKSEENYDKQKQLKFFVSSLLKKDDVINRIENIFI
